# The bark latent fungus *Botryosphaeria dothidea* exacerbates branch dieback following the infection with *Verticillium dahliae*

**DOI:** 10.1007/s44154-026-00288-3

**Published:** 2026-02-10

**Authors:** Ruifeng Guo, Yicheng Li, Chen Tang, Yize Zhao, Mohan Wang, Guanghang Qiao, Steven J. Klosterman, Yonglin Wang

**Affiliations:** 1 State Key Laboratory of Efficient Production of Forest Resources, Beijing Key Laboratory for Forest Pest Control, College of Forestry, Beijing Forestry University, Beijing, 100083 China; 2 Institute of Plant Protection, Beijing Academy of Agriculture and Forestry Sciences, Beijing, 100097 China; 3 United States Department of Agriculture, Agricultural Research Service, Salinas, CA USA

**Keywords:** Verticillium wilt, *Verticillium dahliae*, *Botryosphaeria dothidea*, Microbiota, Co-infection

## Abstract

Verticillium wilt, caused by the soilborne fungus *Verticillium dahliae*, has resulted in high mortality of *Cotinus coggygria* (smoke tree) in China. Symptoms of this disease are complex, many infected smoke trees exhibit wilting or dieback on some branches but no other branches. Whether other microbial taxa act synergistically to contribute to symptom development is unknown. Here, we investigated the microbial community assembly features associated with different branches of smoke trees with or without Verticillium wilt symptoms and established linkages between symptomatic branches and putative keystone taxa. Amplicon data analyses revealed that *V. dahliae* significantly affected the microbiota structure within tree branches. Microbial network connectivity indicated that Verticillium wilt destabilized the network, and fungal communities were more sensitive to Verticillium wilt than the bacterial communities. Based on taxonomic level information, the fungus *Botryosphaeria dothidea* was significantly enriched in diseased branches and positively correlated with the abundance of *V. dahliae.* Through microbial isolations, pathogen co-inoculations, histopathological assays, and RNA-seq analyses, the results indicated that plants infected with *V. dahliae* showed significantly increased susceptibility to *B. dothidea* and downregulated expression of defense-related genes. Overall, the results revealed that Verticillium wilt provokes changes in the structure of the smoke tree microbiome and that these changes likely influence symptom development in some but not all tree branches. The synergistic interplay between the commensal fungus *B. dothidea* and the soil-borne fungus *V. dahliae* promotes wilt progression in smoke trees, offering new insights into developing effective control strategies through fungicides plus enhancing host vigor.

**Supplementary Information:**

The online version contains supplementary material available at 10.1007/s44154-026-00288-3.

## Introduction

Soil-borne plant pathogens cause a range of diseases that threaten the global sustainability of agroforestry (Bailey et al. 2003). *Verticillium dahliae* is a soil-borne pathogenic fungus that causes vascular wilt disease on more than 200 plant species (Klosterman et al. 2009; Fradin and Thomma 2006), such as cotton (Wu et al. 2023), olive tree (Fernandez-Gonzalez et al. 2020), and ornamental tree species such as smoke tree (*Cotinus coggygria* Scop.) (Wang et al. 2013; Li et al. 2024). The disease cycle of *V. dahliae* typically involves three stages: dormant, parasitic, and saprophytic. As initial infection sources, microsclerotia, germinate upon stimulation by host root exudates and form germ tubes. Subsequently, the hyphae penetrate the host plant cells until reaching the vascular tissue, where it produces a large number of conidia. These conidia are carried with the sap stream and trapped in pit cavities or at vessel end walls, so-called trapping sites, where they must germinate and penetrate adjacent vessel elements in order to continue colonization. Sporulation then occurs to start another infection cycle (Fradin and Thomma 2006; Chen et al. 2021). The fungus impairs water flow due to mycelial proliferation and secretome-mediated manipulation of its environment, which can ultimately cause death (Fradin and Thomma 2006; Klosterman et al. 2009).

Smoke trees are highly valued ornamentals that produce the red-leaved forest landscape in Northern China, especially in Beijing. Verticillium wilt poses a serious threat to smoke trees in Beijing, and stands within the forest system have already incurred heavy losses (Wang et al. 2013). In agroecosystems, Verticillium wilt can be effectively suppressed through intercropping, soil fumigation, the selection of disease-resistant varieties, and, in some cases, by controlling environmental conditions. (for example, by adjusting humidity or drainage) (Guo et al. 2024b). However, forest ecosystems can be vastly more complex (Wu et al. 2025a), and some of those control measures cannot be applied because trees are long-lived and there are also site-specific conditions that prevent the use of certain tools like soil fumigation. Thus, efficacious control of Verticillium wilt of smoke tree is difficult due to a multiplicity of factors, and available measures have so far proven unsuccessful.

The plant microbiome is closely associated with plants, encompassing both external and internal compartments of microbes (Wang et al. 2024). The external plant compartments include the root zone, rhizosphere, and rhizoplane below ground, as well as the epiphytic phyllosphere above ground. The external compartment interacts with the surrounding environment, recruiting microorganisms, while internal compartments include the endosphere of both above- and below-ground tissues (Howe et al. 2023). The composition and function of these complex microbial communities directly impact plant growth, health, and performance (Zhang et al. 2025). Their structure and functions evolving in response to stress and environmental stimuli (Liu et al. 2020). Plants and microbes can establish symbiotic relationships under favorable conditions. The recruitment and selection of host-adapted microorganisms is crucial for plant health and nutrition (Zhang et al. 2022; Zancarini et al. 2024; Ping et al. 2024; Wu et al. 2025b). Plant endophytic microorganisms influence growth and development, inhibit competitor growth, and improve stress resistance (Ping et al. 2024). For example, plants respond to various biotic or abiotic stressors, such as pathogen infection, by altering the associated microbial communities (Liu et al. 2021). In addition, interspecific interactions within communities include resource competition, antagonism and metabolic cross-feeding, which can significantly determine microbial functionality (Barber et al. 2022).

Specific plant-beneficial rhizosphere bacteria can suppress plant diseases by directly antagonizing pathogens or by stimulating the plant's innate immune response (Pieterse et al. 2014). Thus, host plants are widely believed to actively regulate the assembly of their microbiomes to enhance their own fitness (Rolfe et al. 2019). When attacked by pathogens, some plants selectively enrich beneficial microorganisms in their rhizosphere to enhance resistance (Gao et al. 2021). For example, when *Astragalus mongholicus* was challenged by *Fusarium oxysporum*, beneficial bacteria such as *Stenotrophomonas*, *Achromobacter*, *Pseudomonas*, and *Flavobacterium* were recruited to the rhizosphere. Additionally, *Stenotrophomonas* sp., *Rhizobium* sp., *Ochrobactrum* sp., and *Advenella* sp. effectively suppressed *Astragalus* root rot (Li et al. 2021). The synergistic interaction between *Streptomyces* and key rhizosphere microorganisms, such as *S. maltophilia* and *P. cellulositrophicus*, significantly enhances the ability of *Streptomyces* to control tomato wilt disease caused by *Ralstonia solanacearum* (Sun et al. 2025). The soilborne fungus *V. dahliae* can manipulate the belowground soil microbiome structure (Fernandez-Gonzalez et al. 2020). Diseases caused by soilborne fungi, including Verticillium wilt, can be suppressed by manipulating the underground microbial community (Yang et al. 2021). Plant endophytic microorganisms influence growth and development, inhibit competitor growth, and improve stress resistance (Ping et al. 2024). Previous studies have mainly examined the microbiota in the soil network (Fernandez-Gonzalez et al. 2020), leaves (Liu et al. 2023), and seeds (Links et al. 2014), and have neglected other plant compartments such as vascular tissues (Zhang et al. 2022). A few studies have reported that some systemic bacteria can spread to above-ground plant compartments through transpiration-driven xylem flow (Zhang et al. 2022). But little is known about the functional relationship between the xylem-inhabiting microbiota and the development of plant diseases, especially in relation to vascular wilt pathogens. Understanding of the microbial communities within the xylem vessels of the smoke tree may be crucial to evaluate their potential influence on health and disease.

The concept of monospecies/monostrain infections is closely tied to the lexicon of plant pathology, where disease epidemics are primarily linked to a single pathogen (Fitt et al. 2006). However, in some plant-pathogen interactions, multiple biotic stresses may affect the plant simultaneously (Wang et al. 2023a). Plant diseases can be caused by multiple pathogenic species, and their co-occurrence is referred to as co-infection (Lamichhane and Venturi 2015). In many cases, a single infecting microbe may not lead to severe disease symptoms, while co-infection with another microbial species may lead to severe disease development due to synergistic interactions (Fitt et al. 2006). Co-inoculation of *Ilyonectria* and *Botryosphaeriaceae* isolates resulted in increased decline of grafted grapevines compared to inoculations of *Ilyonectria* isolates alone (Whitelaw-Weckert et al. 2013). There are also reports of synergistic interactions between differentially aggressive strains of numerous pathogens. For example, *Fusarium* spp. can promote soybean infection by the weakly pathogenic *P. sojae* strains (Wang et al. 2023a). Additionally, plant-associated microbial communities can promote plant nutrient uptake, growth, and resistance to pathogens (Mainwaring et al. 2023). As these studies illustrate, plant pathogens entering a host may encounter not only the host's defenses but also other microbial species within the plant phytobiome that may influence the plant-pathogen interaction (Tollenaere et al. 2016). Symptom-based detection is rarely a reliable means of identifying co-infections, and next-generation sequencing opens up new possibilities for characterizing microbial communities (Tollenaere et al. 2016). The host microbial community may also influence co-infection (Halle et al. 2024). Understanding these community interactions and synergistic infections in plants may be critical to understand microbial pathogenesis and subsequently developing effective disease control strategies.

During a field survey of smoke tree wilt, we observed that large numbers of infected smoke trees showed wilting or dieback of one branch but no symptoms in other branches. To further investigate the underlying mechanism of this phenomenon, we (1) examined the smoke tree-associated microbiome structure in tree branches with and without symptoms, (2) assessed the co-occurrence of interactions between *V. dahliae* and tree branch endophytes, and (3) examined whether the microbiome influences the pathogenesis of *V. dahliae*. Our findings reveal a previously unknown functional trait of the branch endophytic microbiota and provide insight into microbial communities associated with Verticillium wilt of smoke trees, and thus may be useful in devising new approaches for disease control.

## Results

### Verticillium wilt affects the diversity and structure of the endophytic microbial community

**Fig. 1:**
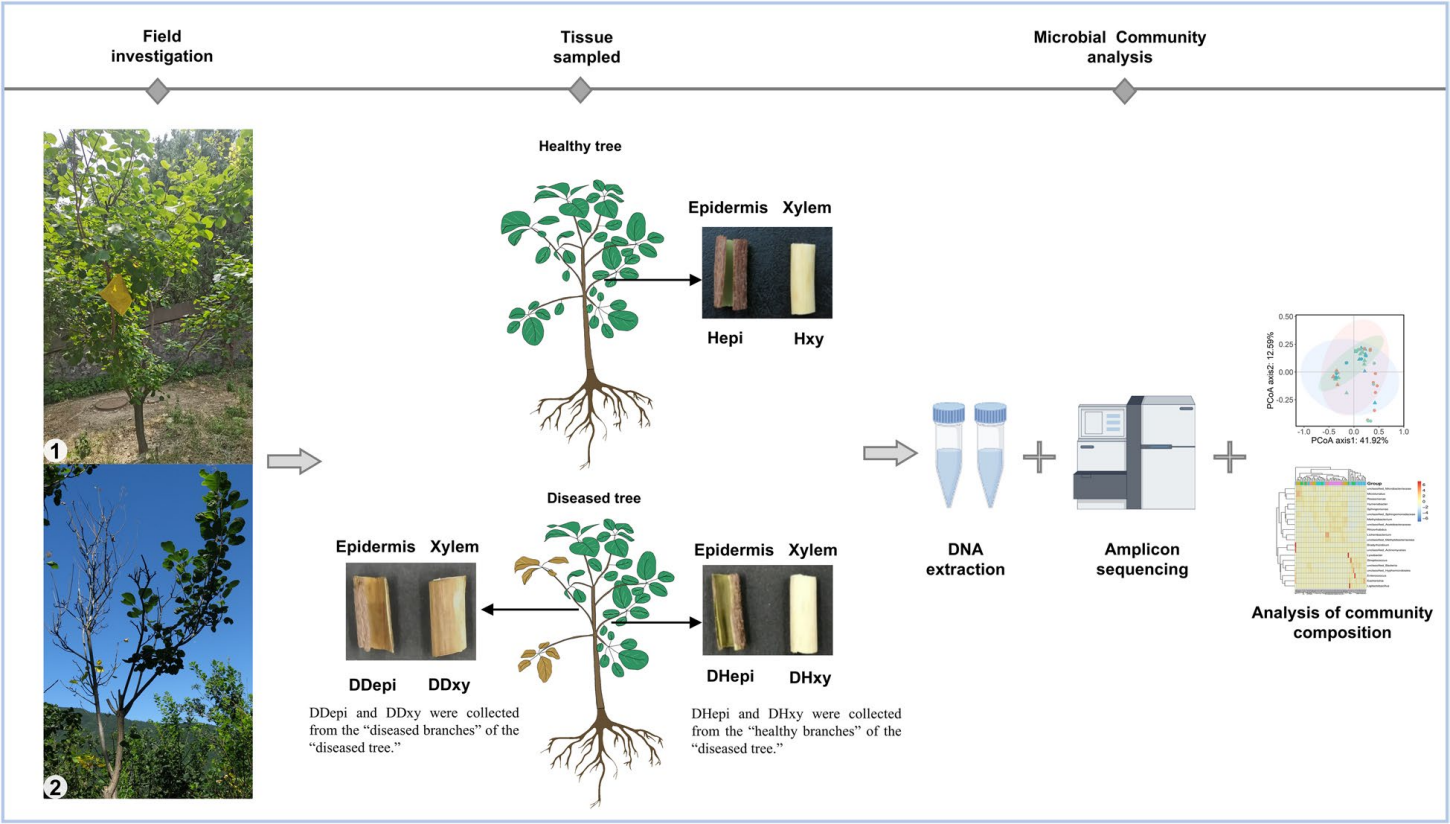
Schematic diagram of sample collection from smoke trees (*Cotinus coggygria*). Hepi: healthy tree branch epidermis; DHepi: diseased tree-healthy branch epidermis; DDepi: diseased tree-diseased branch epidermis; Hxy: healthy tree branch xylem; DHxy: diseased tree-healthy branch xylem; DDxy: diseased tree-diseased branch xylem

**Fig. 2:**
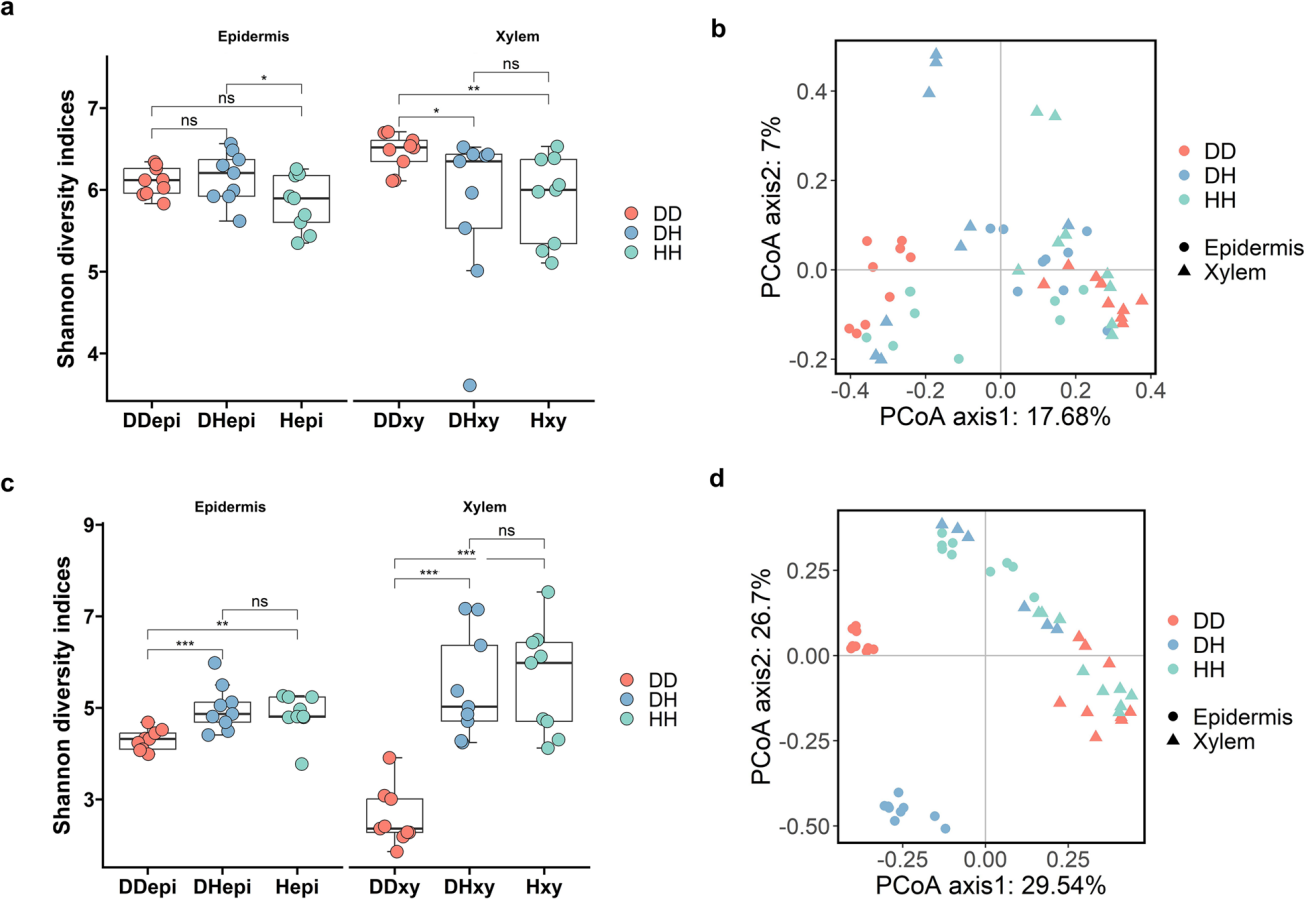
Diversity and structure of bacterial and fungal communities in branches and tissue samples from smoke tree (*Cotinus coggygria*). **a**. Shannon diversity indices of bacteria of HH (green), DH (blue) and DD (red) plants. **b**. Bray–Curtis distance Principal Coordinate Analysis (PCoA) of bacteria.** c**. Shannon diversity indices of fungi of HH (green), DH (blue) and DD (red) plants. **d**. Bray–Curtis distance Principal Coordinate Analysis (PCoA) of fungi. Asterisks denote significant differences between microbial diversity obtained from healthy trees and those trees with Verticillium wilt (*P < 0.05; *P *< 0.01; *P *< 0.001). HH: healthy tree branch; DH: diseased tree-healthy branch; DD: diseased tree-diseased branch; Hepi: healthy tree branch epidermis; DHepi: diseased tree-healthy branch epidermis; DDepi: diseased tree-diseased branch epidermis; Hxy: healthy tree branch xylem; DHxy: diseased tree-healthy branch xylem; DDxy: diseased tree-diseased branch xylem

The 16S rRNA and ITS amplicon sequences were used to investigate the structure of fungal and bacterial communities in healthy tree branches (HH), healthy tree branches from diseased trees infected with *V. dahliae* (DH), and diseased tree-diseased branch (DD), followed by clustering into operational taxonomic units (ASVs; 100% identity) (Fig. 1). The impact of Verticillium wilt on the endophytic microbial communities was conducted by comparing the Shannon diversity index in each compartment (healthy tree branch epidermis (Hepi), diseased tree-healthy branch epidermis (DHepi), diseased tree-diseased branch epidermis (DDepi), healthy tree branch xylem (Hxy), diseased tree-healthy branch xylem (DHxy), and diseased tree-diseased branch xylem (DDxy). For the bacterial communities, the Shannon diversity index revealed that Hepi was the lowest, with no significant difference between DHepi and DDepi; DDxy was significantly higher than DHxy and Hxy (Fig. 2a). In contrast, the Shannon diversity index of fungal communities differed markedly from that of bacterial communities. The Shannon diversity index of DDepi was significantly lower than DHepi and Hepi, and DDxy showed the same trend (Fig. 2c). The respective roles of disease in shaping the microbiota associated with smoke trees were investigated by using Principal Coordinate Analysis (PCoA) and Permutation Multivariate Analysis of Variance (PERMANOVA). Compartments had a greater effect on microbial communities than diseases do on microbial communities (Fig. 2b and Fig. 2d). The differences were significant for both the bacterial community and fungal community composition of healthy and diseased plants (except for the fungal DHxy-Hxy; the bacterial DHxy-Hxy and DDxy-Hxy) (Table S1, S2). To assess the pathogen's impact on the fungal community, we re-analyzed both alpha and beta diversity after excluding *V. dahliae*. Alpha diversity results showed no significant differences in the Shannon index across the compartments (Fig. S1a). In contrast, beta diversity revealed significant differences in fungal community composition between healthy and diseased plants across all compartments, except for the DHxy-Hxy community, which was consistent with the results before *V. dahliae* exclusion (Fig. S1b). Moreover, regardless of *V. dahliae* exclusion, the disease had a greater impact on the fungal community than on the bacterial community (R^2^ = 0.11 for bacteria and R^2^ = 0.40 for fungi, R^2^ = 0.32 for fungi excluding *V. dahliae*, averaged for all compartments) (Table S1, S2, and S3).

**Fig. 3:**
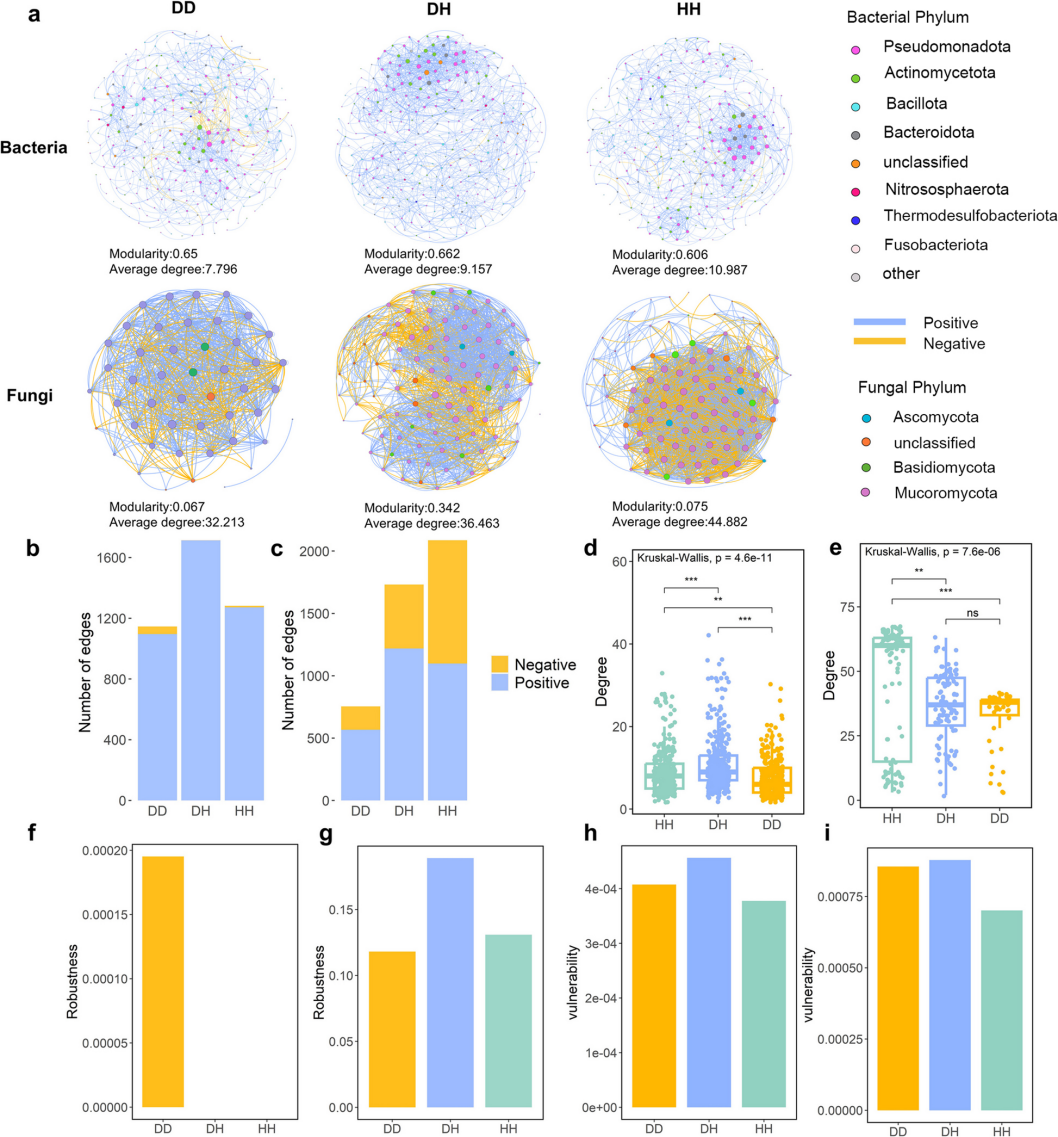
Co-occurrence networks of healthy and diseased smoke trees. **a**. Bacterial and fungal intra-kingdom co-occurrence networks. The nodes are colored according to phylum. Node size indicates the degree of connection. Edge color represents positive (blue) and negative (yellow) correlations. Edges and of bacterial (**b**) and fungal (**c**) networks. Degree of bacterial (**d**) and fungal (**e**) networks. Robustness measured as the proportion of taxa remained with 50% of the taxa randomly removed from each of the bacterial (**f**) and fungal (**g**) networks. Bacterial (**h**) and fungal (**i**) networks vulnerability. Network vulnerability was measured by maximum node vulnerability in each network. HH: healthy tree branch; DH: diseased tree-healthy branch; DD: diseased tree-diseased branch

To investigate whether and how the Verticillium wilt affects the complexity and stability of the molecular ecological networks in smoke tree, we constructed bacterial-bacterial and fungal-fungal intra-kingdom co-occurrence networks. Bacterial-bacterial networks in DD-associated communities had slightly fewer edges and degrees, but a slightly higher proportion of negative edges compared to those in HH (Fig. 3a, b and d). We calculated the robustness of each network by simulating species extinction, which reflected the intensity of interaction between microorganisms. The robustness of the networks showed that DD had the highest robustness (Fig. 3f). The DD and DH bacterial networks possessed a higher vulnerability (the maximum decrease in network efficiency when a single node is deleted from the network) (Fig. 3h) (Yuan et al. 2021). The fungal-fungal intra-kingdom networks showed a sharply different network topology from the bacterial networks. Fungal-fungal networks in DD-associated communities had lower edges and degrees, and a lower proportion of negative edges, compared to fungal networks in HH-associated communities (Fig. 3a, c and e). In addition, the DD fungal network possessed a lower robustness (Fig. 3g). The DD and DH fungal networks possessed a higher vulnerability, which is similar to the bacterial network (Fig. 3h and i). After excluding *V. dahliae* data, the fungal network displayed consistent trends (Fig. S2). The DD fungal network showed low robustness and high vulnerability (Fig. S2), suggesting that the pathogen compromised the network's stability. Fungal networks exhibited greater vulnerability than bacterial networks, indicating that fungal communities are more prone to disease-induced changes (Fig. 3h, i, and Fig. S2). The more complex topological properties of DH bacterial and HH fungal networks suggest stronger microbial interactions. The decline in compositional stability and node connectivity within these communities suggests that the wilt disease significantly disrupts microbial interactions.

### Variation in the composition of endophytic microbial communities

To identify the core microbiota associated with the pathogen, we conducted a comprehensive analysis of the taxonomic composition and relative abundance of bacterial and fungal communities. Phylum level analysis of microbial communities revealed compartment specificity (Fig. S3). Analysis of the bacterial community composition at the phylum level revealed that Pseudomonadota, Bacillota, and Actinomycota were dominant, accounting for more than 80% of all sequences. Bacillota were significantly more abundant in the xylem compartment than in the epidermis compartment (Fig. S3a). In terms of fungal community composition at the phylum level, the Ascomycota were significantly more abundant in the DDepi, DHepi, Hepi, and DDxy than in the DHxy and Hxy compartments (Fig. S3b).

**Fig. 4:**
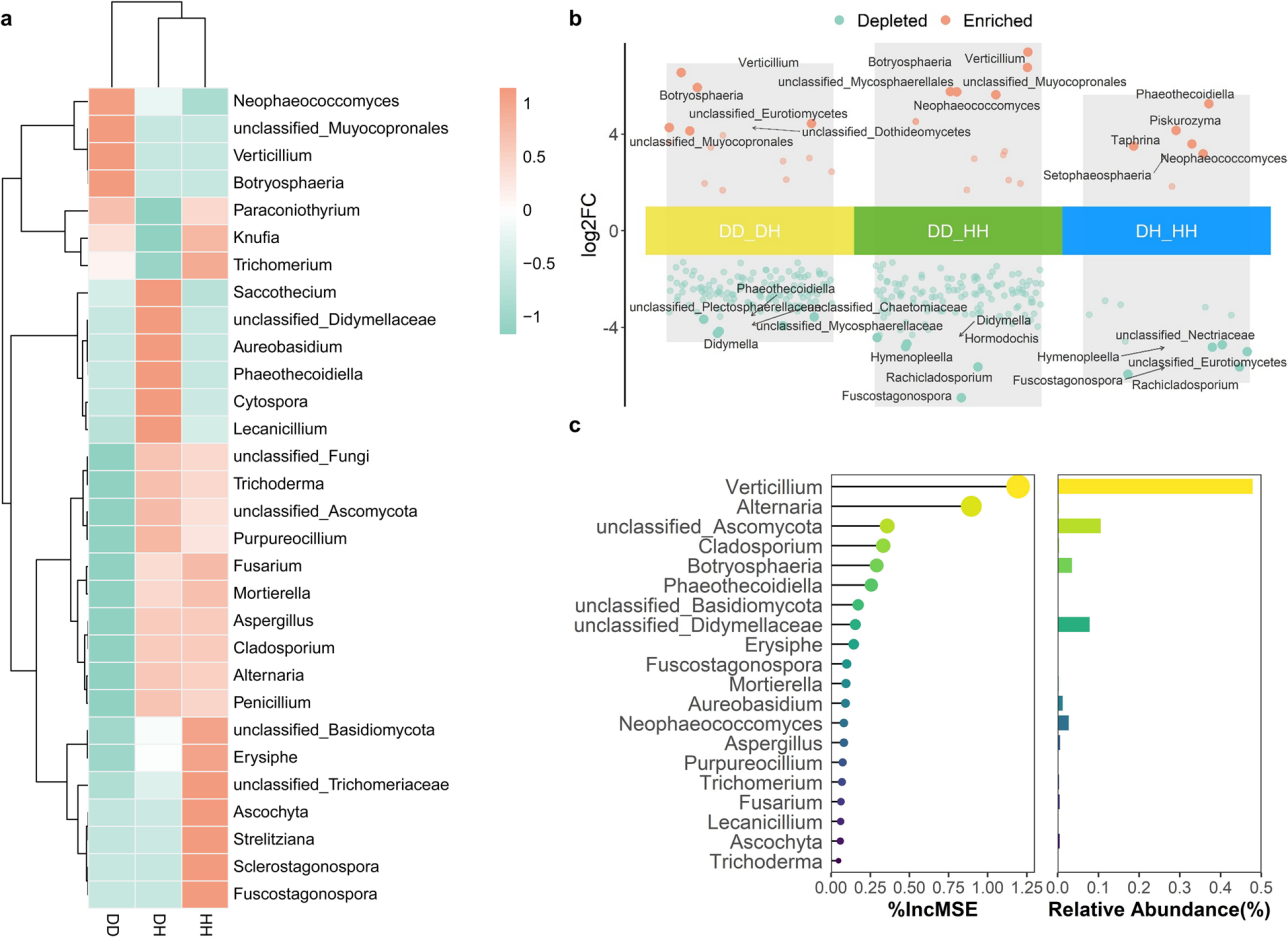
Assembly of fungal communities obtained from healthy and Verticillium wilt diseased branches of smoke trees. **a**. Heat map of the fungal community composition with cluster analysis. Similar samples were clustered horizontally, and vertical patterns illustrate the phylogenetic relationships among the top 30 genera across samples, normalized by rows. **b**. Analysis of the effect of Verticillium wilt of smoke trees (*Cotinus coggygria*) on fungal genus abundance based on volcano plot. Symbols correspond to enrich (red) and depleted (green) genus (*P* < 0.05). **c**. The top 20 most important biomarkers identified by random-forest classification in the DD and the DH group, with the biomarker taxa ranking in descending order of importance in terms of model accuracy. Mean decrease in Gini was used to evaluate the importance level of fungal classes affected by Verticillium wilt. The bar plot showed the relative abundance of the genus in diseased tree-diseased branches. HH: healthy tree branch; DH: diseased tree-healthy branch; DD: diseased tree-diseased branch

We further performed differential analysis on the abundance of taxa associated with diseased and healthy tree branches. The heat map of the top 30 genera revealed that *Verticillium*, *Neophaeococcomyces*, *Botryosphaeria*, and unclassified_*Muyocopronales* were significantly enriched in the DD. The relative abundance of several fungi from the genera *Aureobasidium*, *Phaeothecoidiella* were significantly enriched in the DH. The relative abundance of fungi from the genera *Trichoderma*, *Trichomerium*, *Erysiphe*, *Alternaria*, *Penicillium, Fuscostagonospora*, *Strelitziana* etc. was significantly higher in the HH (Fig. 4a).

Next, we performed differential analysis of the taxa associated with diseased plants and those associated with healthy plants. We screened for significant differences among fungi in the top 5 genera. We further analyzed two-by-two comparisons of DD, DH, and HH. The results showed that *Verticillium* and *Botryosphaeria* were significantly enriched in DD (Fig. 4b). Further random analysis of DD and HH showed that *Verticillium*, *Alternaria*, unclassified_*Ascomycota*, *Cladosporium*, and *Botryosphaeria* were the top genera that significant contributions in the DD, and the relative abundance of *Verticillium*, unclassified Ascomycota, and *Botryosphaeria* was higher in DD (Fig. 4c). We also performed a random forest analysis comparing DD and HH, revealing that *Verticillium* and *Botryosphaeria* exhibited similarly high contribution levels in DD (Fig. S4). We examined the bacterial community using the same method and the bacterial taxon *unclassified_Sphingomonadaceae, Rhizorhabdus, unclassified Actinomycetes*, *Sphingomonas, Methylobacterium,* and *unclassified Acetobacteraceae* were enriched in DD (Fig. S5a). Of these, *Rhizorhabdus* was significantly enriched in DD (Fig. S5b). Random analysis of forest tree branches indicated that *Rhizorhabdus* was potentially the genus contributing to the DD (Fig. S5c). We further found that there were no significant correlations between abundance of *Verticillium* and *Rhizorhabdus* (R^2^ = 0.49, *P* > 0.05). Taken together, the results indicate that Verticillium wilt alters the assembly of endophytic microbial communities in branches.

### Identification of potentially secondary pathogens

**Fig. 5:**
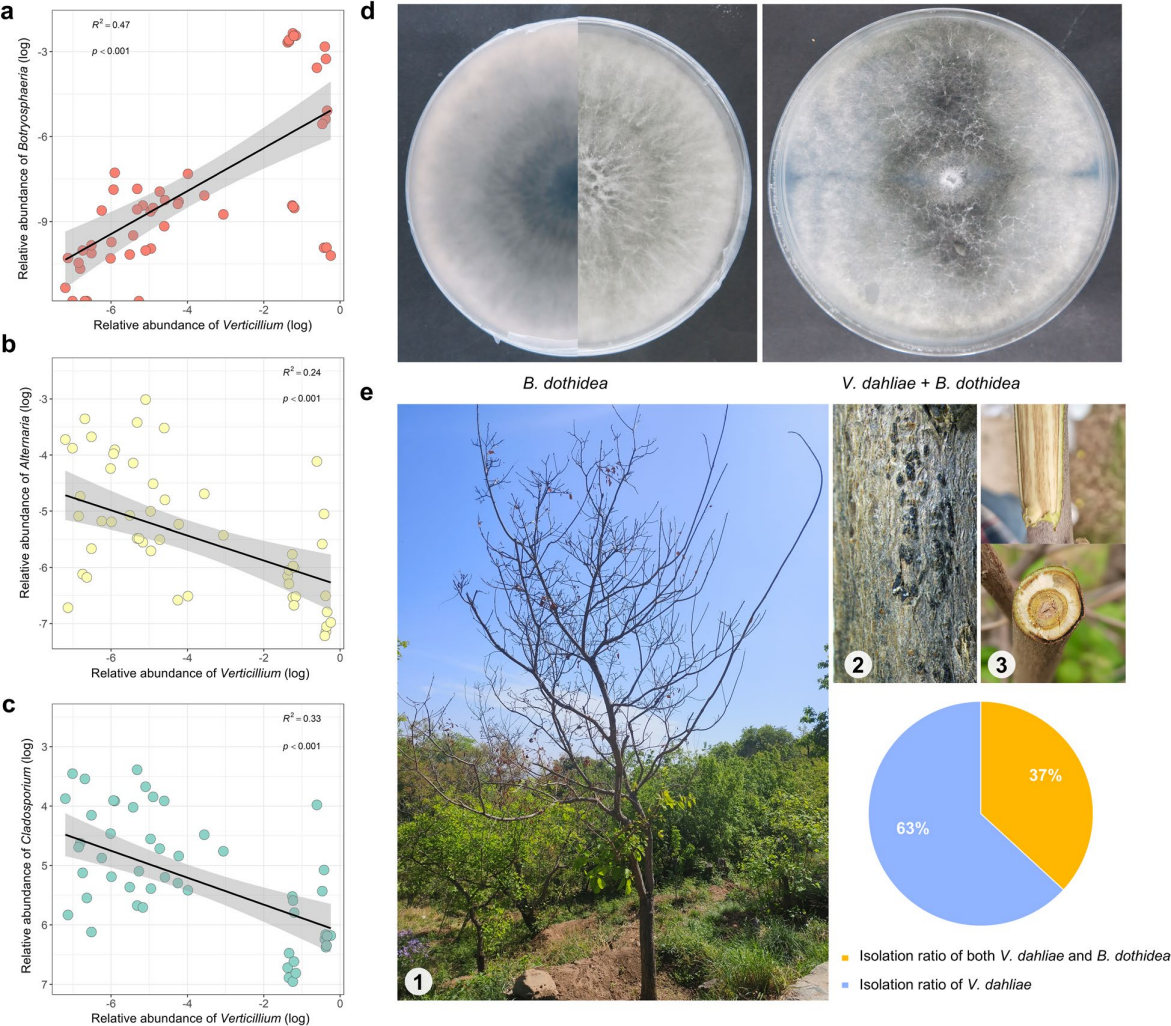
Identification of potential pathogens from smoke trees. **a-c**. Correlation between the relative abundance of *Verticillium* and other fungi (*Botryosphaeria, Alternaria*, *Cladosporium*). **b**. Isolation of *B. dothidea* from diseased branches from smoke tree (*Cotinus coggygria*). **c**. Field surveys and isolation of diseased branches. 1: Diseased smoke tree; 2,3: Diseased branches

To further investigate which genera that significantly contribute to DD are influenced by fungal pathogens, we analyzed the relationship between these microorganisms and the pathogens. Linear analysis showed that the relative abundance of *Verticillium* was negatively correlated with that of *Alternaria*, and *Cladosporium* (Fig. 5a-c), and positively correlated with that of *Botryosphaeria* and the R^2^ for *Botryosphaeria* was higher (R^2^ = 0.47, P < 0.001) (Fig. 5a). We hypothesized that *Botryosphaeria* played a key role in disease development. We isolated and cultured a strain of *B. dothidea* from infected branches, which showed no antagonistic effects against *V. dahliae* (Fig. 5d). Additionally, *B. dothidea* enriched in the bark, while *V. dahliae* enriched in the xylem, suggesting that the two species occupy distinct ecological niches (Fig. S6b).

We investigated the correlation between *B. dothidea* and *V. dahliae* in diseased branches collected from the field. A total of 76 diseased branch samples were collected from Xiangshan, Pofengling, Badaling, and Tongzhou districts, with *V. dahliae* isolated from 63% of the samples and both *B. dothidea* and *V. dahliae* isolated from 37% of the samples (Fig. 5e). These results suggest that *B. dothidea* may play a key role in the development of the disease.

### Co-infection of the smoke tree with *V. dahliae* and *B. dothidea* enhances wilting

**Fig. 6:**
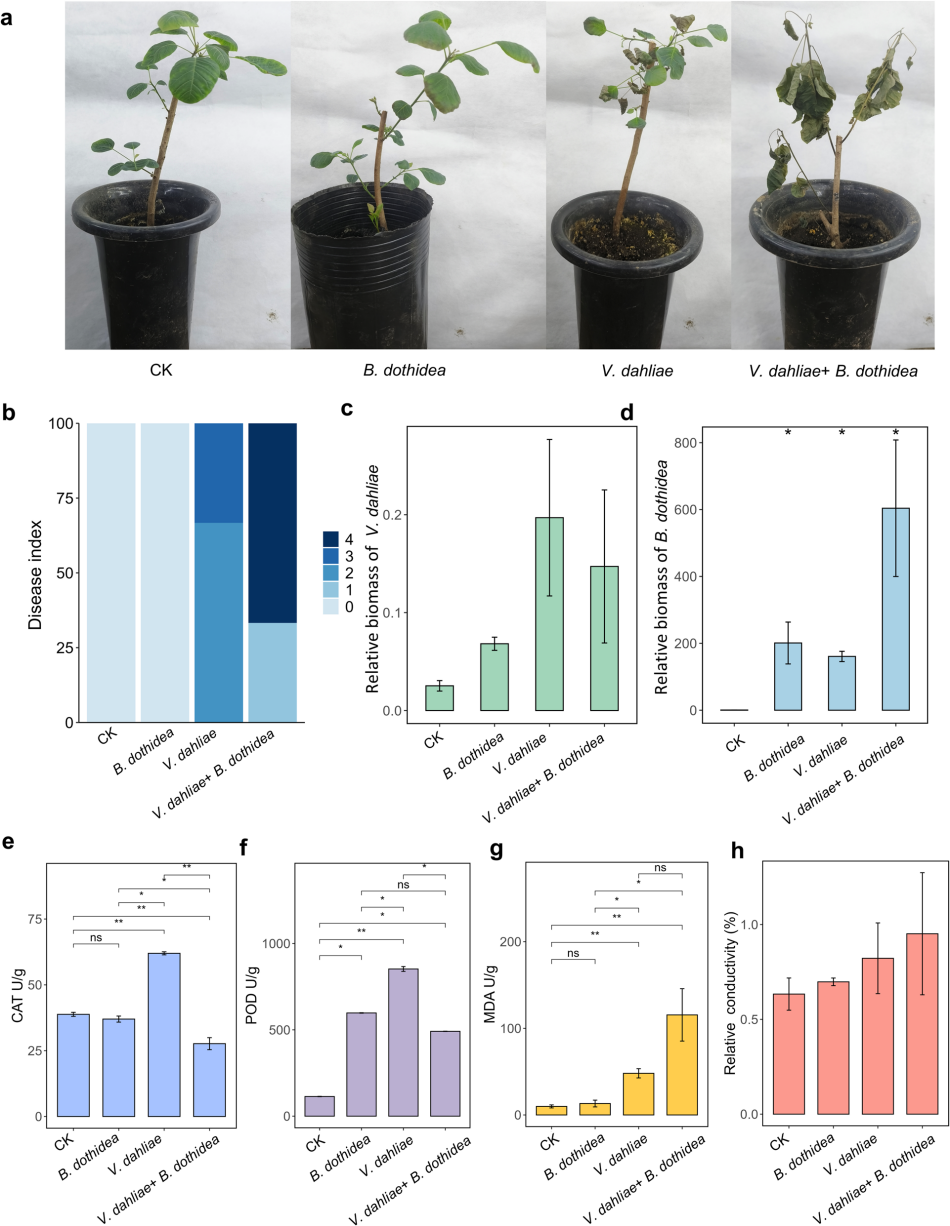
*Botryosphaeria dothidea* infects smoke trees following *Verticillium dahliae* colonization. **a**. Indoor pathogenicity trials. All smoke tree (*Cotinus coggygria*) seedlings were placed in a glasshouse. **b.** Disease indices were scored visually on a scale from 0 (no symptoms) to 4 (completely wilted or dead). Bars represent the percentages of plants within each disease index class. **c.** Quantification of *Verticillium dahliae*. DNA of smoke tree under different treatments was subjected to qPCR to estimate pathogen biomass. The relative quantitative method (2 ^−ΔΔCt^) was used to evaluate the quantitative variation. **d**. Quantification of *B. dothidea*. DNA of smoke tree under different treatments was subjected to qPCR to estimate pathogen biomass. The relative quantitative method (2 ^−ΔΔCt^) was used to evaluate the quantitative variation. **e**. Catalase **(**CAT) activity. **f**. Peroxidase (POD) activity. **g**. Malondialdehyde (MDA) content. **h**. Relative conductivity (REC) in samples from *V. dahliae*-infected plants. ∗*P* <0.05; *P* < 0.01.Error bars: ± standard deviation with five biological replicates

To investigate the effects of *B. dothidea* and *V. dahliae* on smoke tree, we conducted pot experiments in the greenhouse. The results showed that inoculation with *V. dahliae* followed by inoculation with *B. dothidea* yielded the most severe wilt symptoms. In contrast, inoculation with *B. dothidea* alone did not cause disease (Fig. 6a and b). We further performed quantitative real-time PCR (qPCR) analyses to gain deeper insights into the abundance of *V. dahliae* and *B. dothidea* in the plants. The results showed that the levels of *V. dahliae* were not significantly different between treatments (Fig. 6c). The biomass of *B. dothidea* was significantly increased in the co-inoculation of *V. dahliae* and *B. dothidea*. (Fig. 6d).

To investigate how *B. dothidea* becomes pathogenic and its synergistic mechanisms with *V. dahliae*, we compared smoke trees inoculated with *V. dahliae* alone to those co-inoculated with *V. dahliae* and *B. dothidea* to assess the symptoms exhibited by the infected trees. During plant-pathogen interactions, plants rapidly generate ROS including hydrogen peroxide (H_2_O_2_) (Tang et al. 2020a). We measured the activity of antioxidant enzymes. Catalase (CAT) and peroxidase (POD) are essential antioxidant enzymes for scavenging ROS. In the *V. dahliae* inoculation group, CAT and POD activities were significantly higher than in the *B. dothidea* single inoculation and *V. dahliae* + *B. dothidea* co-inoculation groups. Additionally, CAT activity was lowest in the *V. dahliae* + *B. dothidea* co-inoculation group (Fig. 6e and f). We further measured the relative conductivity (REC) and malondialdehyde (MDA) content to investigate the degree of cell membrane damage in the *V. dahliae* inoculation treatment. The results indicated that plants in the *V. dahliae* + *B. dothidea* inoculated plants had the highest MDA, followed by the *V. dahliae* inoculated plants (Fig. 6g). No significant differences in REC were observed among treatment groups; however, plants inoculated with *V. dahliae* + *B. dothidea* exhibited the highest REC levels, which aligned with the MDA trend (Fig. 6h). These results indicate that *B. dothidea*, an opportunistic pathogen, transitions from an endophyte to a pathogenic fungus following *V. dahliae* infestation.

### Infestation by *V. dahliae* reduced plant defense responses

**Fig. 7:**
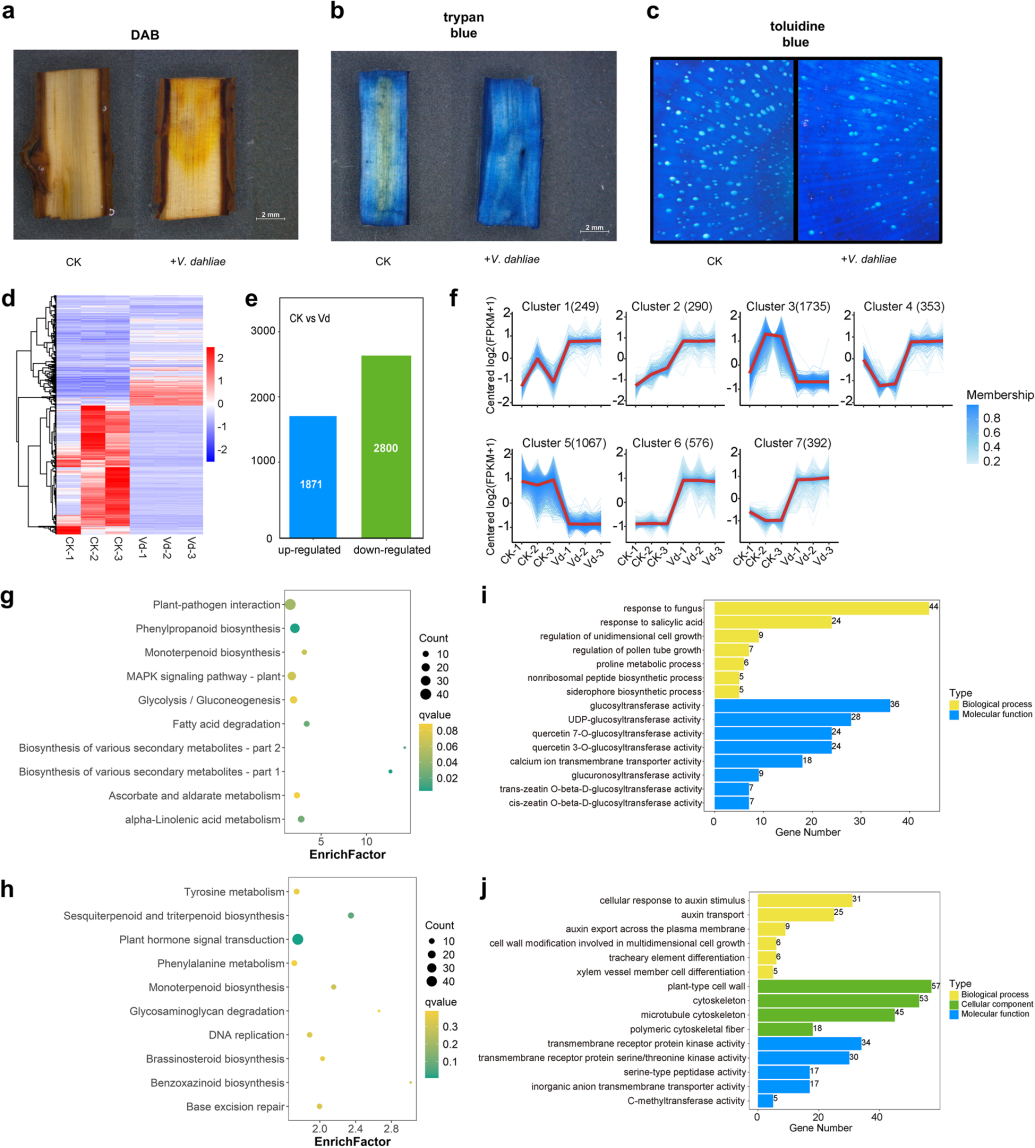
Transcriptome profiles of smoke tree branches infected with *Verticillium dahlia*. a. 3,3’-diaminobenzidine (DAB) staining of branch segments from *V. dahliae*-infected plants. CK is the control. **b**. Trypan blue staining of branch segments. CK is the control. **c**. Observations of xylem vessel clogging from *V. dahliae*-infected plants. CK is the control. **d**. Cluster analysis showed that the patterns of differentially expressed genes (DEGs) were similar among three biological replicates. **e**. Differentially expressed genes in the *V. dahliae*-infected in smoke tree (*Cotinus coggygria*) tissues compared with CK with adjusted *P* < 0.05. **f**. Trend clustering of the expression levels of all DEGs resulted in 7 distinct expression patterns. In the graph, the red lines represent the overall expression trend of each pattern. KEGG enrichment analysis of upregulated (**g**) and downregulated (**h**) differentially expressed genes. Scatter plots are created to display the top 10 most significant KEGG pathways. In the graph, the horizontal axis represents the level of significance of pathway enrichment, and the vertical axis represents the KEGG pathways. The size of the dots indicates the number of genes annotated to each KEGG pathway. GO enrichment analysis of upregulated (**i**) and downregulated (**j**) differentially expressed genes

ROS accumulation has been considered an important factor in maintaining the pathogenicity of necrotrophic phytopathogens. To detect H_2_O_2_ accumulation, we performed 3,3’-diaminobenzidine (DAB) staining of branch segments from the *V. dahliae*-inoculated treatment and *V. dahliae*-uninoculated treatment, and histochemical analyses indicated *V. dahliae*-inoculated treatment accumulated more H_2_O_2_ than the *V. dahliae*-uninoculated treatment (Fig. 7a). We also performed trypan blue staining on the plants following treatments of *V. dahliae*-inoculated and *V. dahliae*-uninoculated to observe the viability of the branches. Trypan blue staining revealed that the *V. dahliae*-inoculated treatment had more dead cells than the *V. dahliae*-uninoculated treatment (Fig. 7b). We sampled and chemically stained the smoke tree tissue sections and analyzed them using microscopy. We observed that there was solid vessel blockage in the *V. dahliae*-inoculated treatment (Fig. 7c). These results suggest that *V. dahliae* infection disrupts the plant's defense system.

To better understand the changes at the molecular level in *V. dahliae*-inoculated plants, we performed RNA-seq analysis on branches infected with *V. dahliae* and CK treatments. Cluster analysis of differentially expressed genes (DEGs) showed sets of genes with similar expression patterns which clustered together in three biological replicates. Differential expression analysis revealed that 2800 predicted genes were significantly downregulated and 1871 predicted genes were significantly upregulated in the *V. dahliae*-inoculated plants compared with the CK (Fig. 7d). They were manually divided into 7 clusters according to their expression pattern (Fig. 7e). Notably, a total of 1067 genes in cluster 5 were significantly downregulated in the diseased branches. Functional annotation of these genes revealed that 394 of them were associated with plant defense, including plant disease resistance genes, protein kinases, jasmonic acid-responsive, chitinase-binding proteins, terpenoid synthases, cellulose synthases, cysteine and serine protease inhibitors, redox regulators, transcription factors, and others (Fig. 7f and Table S4).

We analyzed the DEGs by KEGG analysis and found that the most important enriched pathways were involved in plant-pathogen interactions, phenylpropanoid biosynthesis, MAPK signaling pathways—plant, glycolysis/gluconeogenesis, alpha-linolenic acid metabolism, ascorbate and aldarate metabolism, fatty acid degradation, monoterpenoid biosynthesis, and biosynthesis of various secondary metabolites (Fig. 7g). In addition, plant hormone signal transduction, sesquiterpenoid and triterpenoid biosynthesis, tyrosine metabolism, phenylalanine metabolism, base excision repair, DNA replication, monoterpenoid biosynthesis, brassinosteroid biosynthesis, benzoxazinoid biosynthesis and glycosaminoglycan degradation were significantly downregulated after infestation with *V. dahliae* (Fig. 7h).

Gene ontology (GO) annotation showed that the significantly upregulated genes were involved in many biological processes, such as response to fungus (GO:0009620), siderophore biosynthetic process (GO:0019290), proline metabolic process (GO:0006560), nonribosomal peptide biosynthetic process(GO:0019184), response to salicylic acid (GO:0009751), and regulation of pollen tube growth (GO:0080092). The molecular function analysis revealed that they were mainly related to the glucosyltransferase activity (GO:0046527), cis-zeatin O-beta-D-glucosyltransferase activity (GO:0050502), trans-zeatin O-beta-D-glucosyltransferase activity (GO:0050403), glucuronosyltransferase activity (GO:0015020), quercetin 3-O-glucosyltransferase activity (GO:0080043), quercetin 7-O-glucosyltransferase activity (GO:0080044), UDP-glucosyltransferase activity (GO:0035251), calcium ion transmembrane transporter activity (GO:0015085) (Fig. 7i). Similarly, significantly down-regulated genes included biological processes, cellular components, and molecular functions. Downregulated DEGs were those mainly associated with auxin transport (GO:0060918) and cellular response to auxin stimulus (GO:0071365). As for the cellular component cluster, most downregulated DEGs were attributed to plant-type cell wall (GO:0009505), microtubule cytoskeleton (GO:0015630), cytoskeleton (GO:0005856) and the molecular function division was mainly concentrated in transmembrane receptor protein kinase activity (GO:0019199), transmembrane receptor protein serine/threonine kinase activity (GO:0004675), and others (Fig. 7j).

We also performed RNA-seq analysis of tissues obtained from *B. dothidea*-infected branches, which revealed a significant downregulation of pathways involved in plant hormone signal transduction, tyrosine metabolism, glycosaminoglycan degradation, DNA replication, and the biosynthesis of brassinosteroids and monoterpenoids. These results are consistent with those observed in *V. dahliae*-infected branches (Fig. S7a). The most significant enriched pathways of plant-pathogen interactions and phenylpropanoid biosynthesis were consistent with those observed following *V. dahliae* infection (Fig. S7b). In addition, transmembrane receptor protein kinase activity (GO:0019199) and calcium ion transmembrane transporter activity (GO:0015085) were among differentially down- and up-regulated genes in *B. dothidea*-infected branches, respectively.

**Fig. 8:**
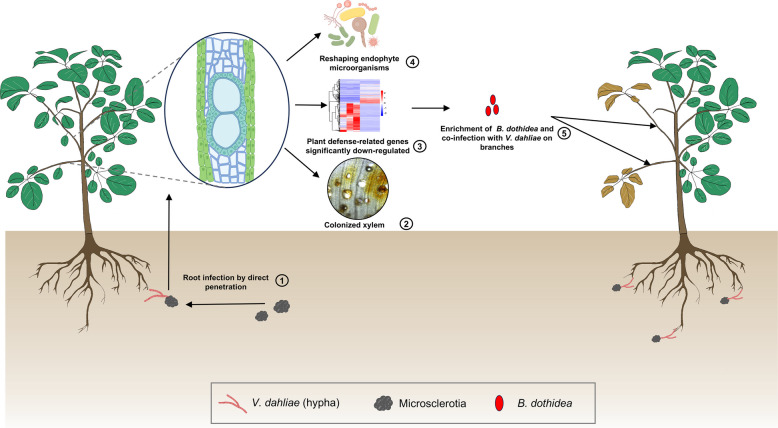
Schematic model illustrating the synergistic action of *Verticillium dahliae* and *Botryosphaeria dothidea* in promoting wilt disease of smoke trees. The soilborne microsclerotia of *V. dahliae* germinate and infect the smoke tree (*Cotinus coggygria*) roots (1). Colonization of the xylem tissue and by *V. dahliae* (2) causes significant down-regulation of plant defense-related genes, which in turn (3) causes a shift in endophyte microorganisms (4) such as *B. dothidea* (5) that act synergistically with *V. dahliae* to exacerbate Verticillium wilt symptoms

## Discussion

In this study, we revealed disease-induced changes in the diversity and composition of endophytic microbial communities in smoke trees. One of these endophytes identified, *B. dothidea*, was further investigated due to its known role as an opportunistic pathogen in other plant-pathogen interactions (Aguirre et al. 2024). We discovered a synergistic interaction between *V. dahliae* and the opportunistic pathogen *B. dothidea* (Aguirre et al. 2024), which promotes Verticillium wilt symptom severity. The results of the RNA-seq analyses further indicated a reduction in expression of genes encoding defense-related pathways and enzymes in *V. dahliae*-infected smoke tree interactions, which may further promote pathogenicity of both *B. dothidea* and *V. dahliae* in smoke trees (Fig. 8).

The marked changes in the diversity and composition of microbial communities are closely linked with crop growth and yield. (Bastida et al. 2021). In this study, the highest α diversity index of diseased branches was found in the bacterial community, while the lowest α diversity index of diseased branches was found in the fungal community. Cardoni et al. (2023) showed that the α diversity index was increased after inoculation with *V. dahliae*. This may be because plants recruit microorganisms to fight the disease at the time of infection, causing an increase in the α diversity index. It is plausible that as disease severity increases, plant microbiome α diversity decreases. β diversity suggests that disease has a greater effect on the endophytic fungal microbial community of branches than on the endophytic bacterial microbial community. Microbes form a complex and diverse co-occurring network through direct or indirect interactions, shaped by interspecies relationships such as symbiosis and competition. Microbiome interactions extend beyond microbial communities to include interactions between microbes and their hosts, where they play critical roles in host development, metabolism, homeostasis, and immunity (Layeghifard et al. 2017). Disturbances or imbalances in the host-associated microbiome, commonly termed dysbiosis, are associated with adverse host responses and can lead to severe pathologies (Layeghifard et al. 2017). In this study, fungal networks exhibited greater vulnerability than bacterial networks, suggesting that fungal communities are more susceptible to disease disturbances. Analyses of Verticillium wilt of olive revealed that the disease can have a significant effect on microbial community structure (Fernandez-Gonzalez et al. 2020). Additionally, previous studies have shown that disease affects endophytic fungal network communities in pepper stems more than bacterial communities, and that fungal communities are more sensitive to disease (Gao et al. 2021). These results are consistent with our findings.

Previous studies have demonstrated that differences in microbial community composition can be linked to plant growth promotion potential (Wang et al. 2023b). We observed large changes in the relative abundance of dominant microbial taxa. This result is consistent with previous findings that microbial communities have a dynamic influence on plant growth and health (Guo et al. 2024a). In the present study, the relative abundance of fungi from the genera *Trichoderma, Penicillium,* and *Strelitziana* etc., was significantly higher in the healthy branches. Wu et al. (2025c) isolated three *Penicillium* strains from soil, all of which demonstrated antagonistic activity against the *Phytophthora capsici* and induced disease resistance in pepper plants. *Trichoderma* is a well-known plant growth promoting microorganisms and biological control agent. In accordance with Wang et al. (2017), beneficial microbes harbored in healthy soil can improve plant growth and control soilborne diseases. Furthermore, using plant probiotics to combat plant diseases has been proposed as a sustainable approach for pathogen control and for supporting plant performance (Xu et al. 2024). Isolation, culture, and inoculation of fungal species belonging to the genera *Trichomerium*, *Erysiphe*, *Alternaria*, and *Fuscostagonospora* are the only ways to confirm their positive effect on disease tolerance in smoke trees and to further investigate the underlying mechanisms that mediate these effects.

*B. dothidea* is one of the most widespread opportunistic pathogens to cause a canker and dieback of many woody plants worldwide (Marsberg et al. 2017). *V. dahliae* is a systemic invasive pathogen (Fradin and Thomma 2006), while *B. dothidea* is an opportunistic pathogen and one that causes disease when triggered with plant stresses, such as drought, physical damage, and unsuitable growing environments (Marsberg et al. 2017; Aguirre et al. 2024). In the last few decades, *B. dothidea* has been recognized primarily as endophytes that infect healthy tissue of woody plants and remains dormant until the weakened vigor during the onset of stressful conditions (Sakalidis et al. 2011). It is clear that once host vigor was high, *B. dothidea* incubates within bark but not cause significant tissue damage or disease symptoms (Aguirre et al. 2024).

When plants are infected with fungi, a surge in ROS typically triggers the plant defense response (Li et al. 2025). Excessive accumulation of ROS causes severe peroxidative damage (oxidative stress) to plant cell membranes and biological systems (Fu et al. 2023). Antioxidant enzymes, such as SOD and POD, act as ROS scavengers to maintain ROS homeostasis (Yang et al. 2025). In our study, DAB staining revealed that smoke tree accumulated significant amounts of ROS under pathogen-induced *V. dahliae* stress. Moreover, our results revealed that the expression of genes involved in plant hormone signal transduction, tyrosine metabolism, glycosaminoglycan degradation, DNA replication, and the biosynthesis of brassinosteroids and monoterpenoids was significantly downregulated in smoke tree infested by *V. dahliae*. The plant hormones salicylic acid, jasmonate, ethylene, and auxin play central roles in plant resistance to pathogens, including *V. dahliae*, via defense pathway-related signal transduction (Berendsen et al. 2018). Benzoxazinone glucosides are defensive metabolites that are produced constitutively from indole-3-glycerol phosphate, the precursor of Trp (Singh et al. 2023). Brassinosteroids play vital roles in regulating plant growth and stress responses (Nolan et al. 2017). Terpenoids have been considered to play important roles in biotic interactions, including attraction of beneficial organisms and by providing defense against insects and pathogens (Singh et al. 2023). *V. dahliae*-induced plant stress responses and RNA sequencing analysis indicate that plant defense responses are significantly impaired following infection with *V. dahliae*.

We observed that *B. dothidea* was enriched in diseased branches and exhibited a significant positive correlation with *V. dahliae*. We isolated *B. dothiea* and found that co-inoculation with *B. dothidea* accelerated plant wilting. This result is consistent with the findings of Aguirre et al. (2024), who conducted an extensive study of the ulcerative fungi *B. dothiea* and found that when host vigour is high, *B. dothiea* does not cause significant tissue damage or disease symptoms. This aligns with our findings: when inoculated solely with *B. dothiea*, the host does not exhibit wilting symptoms. Overall, the results demonstrate that Verticillium wilt induces changes in the structure of the smoke tree microbiome, leading to significant biotic stress in the plants. This stress triggers the transformation of *B. dothidea* from an endophytic fungus to an opportunistic pathogen, thereby accelerating the progression of wilting in smoke trees.

## Conclusion

In conclusion, we identified *B. dothidea* as a key microbe that promotes Verticillium wilt disease progression in smoke trees by acting synergistically with *V. dahliae*. These findings may offer new strategies for controlling wilt, and studies are currently underway in smoke tree plots to assess the effectiveness of plant immune activators to reduce disease caused by co-infections of *V. dahliae* and *B. dothidea*.

## Materials and Methods

### Sample collection

All samples were collected from smoke trees at Xiangshan Park (39°59′ N, 116°10′ E), Beijing, China. Smoke trees that displayed no wilt symptoms were classified as healthy; trees that showed wilting and brown vascular bundle symptoms were classified as diseased. Twelve trees (6 healthy and 6 diseased, respectively) were randomly selected for sampling. The diseased and healthy branches of the diseased trees were collected. Three samples of healthy branches and three samples of diseased branches were collected from each diseased tree. Three samples of healthy branches were also collected from each healthy tree. After collection, the samples were placed in sealed bags, transported to the laboratory immediately, and stored in a −80℃ freezer.

### DNA extraction and amplicon sequencing

The smoke tree stem samples were divided into the epidermis and xylem (the epidermis was separated from the xylem using sterile blades), accordingly (Fig. 1). In total, 54 samples were obtained and labeled as epidermis or xylem as the two compartments in the analyses. Thus, the experimental design consisted of 2 compartments × 3 biological replicates × 3 technical replicates × 3 health states (Healthy branches from Healthy trees (HH); Healthy tree branches from Diseased trees (DH); Diseased branches from Diseased trees (DD)). For each replicate, a composite contained two mixed samples. All root and soil samples were stored at −80 °C until DNA extraction.

Total DNA was extracted from samples using a TGuide S96 Magnetic Soil/Stool DNA Kit (Tiangen Biotech, Beijing) following the manufacturer’s protocol. The DNA quality was checked by agarose gel electrophoresis, and the DNA concentrations were determined by using a Nanodrop (ThermoFisher Scientific) spectrophotometer.

The primers 338 F (5'-ACTCCTACGGGAGGCAGCA-3') and 806R (5'- GGACTACHVGGGTWTCTAAT-3') were used to amplify the V3–V4 region of bacterial 16S rRNA, and the primers ITS1F (5'-CTTGGTCATTTAGAGGAAGTAA-3') and ITS1R (5'-GCTGCGTTCTTCATCGATGC-3') were used to amplify the ITS1 of fungi. Both the forward and reverse 16S and ITS1 primers were tailed with sample-specific Illumina index sequences to allow for deep sequencing. The PCR was performed in a total reaction volume of 10 μl: DNA template (5–50 ng), forward primer (10 μM) 0.3 μl, reverse primer (10 μM) 0.3 μl, KOD FX Neo Buffer 5 μl, dNTP (2 mM each) 2 μl, KOD FX Neo 0.2 μl, and finally ddH_2_O up to 20μL. After initial denaturation at 95 °C for 5 min, followed by 20 cycles of denaturation at 95 °C for 30 s, annealing at 50 °C for 30 s, and extension at 72 °C for 40 s, and a final step at 72 °C for 7 min. The amplified products were purified with Omega DNA purification kit (Omega Inc., Norcross, GA, USA) and quantified using Qsep-400 (BiOptic, Inc., New Taipei City, Taiwan, ROC). The amplicon library was paired-end sequenced (2 × 250) on an Illumina novaseq6000 (Beijing Biomarker Technologies Co., Ltd., Beijing, China). The original image data files were converted into original sequenced reads by base calling analysis, and the results are stored in the FASTQ (fq) file format.

### Amplicon sequencing data processing

The raw reads from the sequencing were filtered using TRIMMOMATIC v0.33 (Bolger et al. 2014), and the primer sequences were identified and removed using CUTADAPT 1.9.1 (Kechin et al. 2017) to obtain clean reads. The clean reads for each sample were spliced by overlap using USEARCH v10, and the spliced data were filtered for length. Chimeric sequences were identified and removed using UCHIME v8.1 software (Edgar et al. 2011). Also, sequences identified as mitochondrial, chloroplastic, and unknown (unclassified at the kingdom level) were removed from the dataset to obtain the final reads for analyses. The DADA2 (Callahan et al. 2016) algorithm was used to quality filter and generate amplicon sequence variations (ASVs) and a feature table after the primer sequences were deleted. Taxonomic assignment was performed using NCBI reference databases for bacteria and fungi. The taxonomic annotation of the feature sequences was carried out using a simple Bayesian classifier combined with a comparative method to obtain the taxonomic information of the species corresponding to each feature, and the community composition of each sample was counted at each level (phylum, class, order, family, genus, species). A table of species abundance at different taxonomic levels was generated using QIIME2 (Bolyen et al. 2019).

### Fungal infection assays

Smoke tree seedlings (approximately 20 cm in height) were grown in a greenhouse at Beijing Forestry University (25℃, 8 h dark/16 h light cycle). The six treatments were set up as CK (control; untreated), inoculated with *B. dothidea* alone, inoculated with *V. dahliae* alone, and co-inoculated with *V. dahliae* plus *B. dothidea*. Each treatment group consists of five replicates. Both fungal strains were cultured on potato dextrose agar (PDA) medium. After surface sterilization, the stem bases were wounded and inoculated with a 5-mm diameter PDA plug covered with *B. dothidea* mycelium. A root-dip method was used for the *V. dahliae* inoculations (Tang et al. 2020b).

### Relative electrical conductivity measurements

The smoke tree branches were sampled from the same growth stage and a similar morphological position for the control and the *V. dahliae*-inoculated treatments. The samples were cut into 2 mm sections. This was approximately 0.1 g per section. The samples were placed in test tubes containing 6 mL of deionized water, mixed thoroughly, and shaken well after 12 h extraction at room temperature. There were three replicates per treatment. The relative electrical conductivity (REC) measurements were conducted using a DDS-11 (Leici-DDS-11A, Shanghai, China) and calculated according to the formula (Fu et al. 2023): relative ion leakage = C1/C2 × 100% (C1 represents the pre-boiling water bath conductivity and C2 represents the post-boiling water bath conductivity).

### ROS analyses and trypan blue staining

The integrity of the cell membrane and cell survival were examined by trypan blue staining. Plant branches were cut into 2 mm sections and immersed in a trypan blue solution for 1h. The sections were destained in 96% ethanol overnight and photographed.

To detect the production of H_2_O_2_, plant branches were cut into 2 mm sections and immersed in 3,3'-diaminobenzidine (DAB) solution overnight, and placed in 96% ethanol and decolorized overnight.

### Determination of Catalase, peroxidase and malondialdehyde

Assays were conducted using plant catalase (CAT), plant peroxidase (POD), and plant malondialdehyde (MDA) kits (all from Nanjing Jiancheng Bioengineering Institute, Nanjing) following the manufacturers' instructions.

### Histological analyses

Smoke tree stem internodes were cut into 2 mm sections, and incubated in ethylene glycol ethyl ether acetate I (37 ^◦^C, 6 h) and ethylene ether acetate II overnight (37 ^◦^C). Then, the internodes were placed in ethylene glycol ethyl ether acetate III (10–15 min at room temperature) and ethylene glycol ethyl ether acetate IV (10–15 min at room temperature). The internode sections were rinsed with running water and placed in toluidine blue staining solution (∼2 min), and washed. Photographs were taken under microscopy (ZEISS Stemi-508).

### Sample preparation for RNA‑Seq analysis

To prepare samples for transcriptome sequencing after infestation with *B. dothidea*, the isolated smoke tree branches were cut into 5 cm long branch segments, rinsed with distilled water, then rinsed clean with sterile water and air-dried. A small clump of freshly grown mycelium, approximately 1 cm in diameter and cultured in PDB liquid medium for 3 days, was inoculated onto the burn site, which was then wrapped in bark to increase the contact area between the mycelium and scalded wounds. The inoculated branches were placed at 25 °C for incubation. Bark and xylem from the lesion were collected from smoke tree branches at 0 dpi and 5 dpi, quickly frozen in liquid nitrogen. Each treatment consisted of three replicates. Each replicate consisted of a composite containing two mixed samples. The samples were ground into powder, and total RNA was extracted using the RNA Easy Fast Plant Tissue Kit (majorbio, China) according to the manufacturer's protocol.

### Transcriptome sequencing and bioinformatic analysis

The libraries were prepared using an Illumina Truseq™ RNA Prep Kit. The libraries were sequenced on an Illumina HiSeq platform, and 150 bp paired-end reads were generated. Data were filtered to remove adapters. The paired-end clean reads were aligned to the reference genome using Hisat2 v. 2.0.5. Differential gene expression analysis was conducted using DESeq2 and the screening criteria were padj < 0.05 and fold change ≥ 2 or ≤ − 2 (WT versus mutant). Gene Ontology (GO) and Kyoto Encyclopedia of Genes and Genomes (KEGG) functional enrichment analysis of the differential gene sets was performed using the cluster Profiler software (Kanehisa and Goto 2000).

### Co-occurrence network analysis

Bacterial and fungal networks were constructed separately for each treatment. Co-occurrence patterns were reconstructed by calculating the ASV-level abundance matrix-based Spearman's rank coefficients. Only robust and significant correlations (|r|> 0.6, *P* < 0.05) between ASVs were selected for network construction. The network was visualized using the Gephi v0.9.6 software (Bastian et al. 2009). Topological characteristics including the number of positive and negative edge correlations, average connectivity, and modularity were calculated for all co-occurrence networks. Robustness of a network is defined as the proportion of the remaining species in this network after random or targeted node removal. For simulations of random species removal, a certain proportion of nodes was randomly removed (Yuan et al. 2021). The vulnerability of each node measures the relative contribution of the node to the global efficiency (Yuan et al. 2021).

### Statistical analysis

Alpha diversity (Shannon indices) indices of bacterial and fungal communities were calculated in QIIME2 (Bolyen et al. 2019). The differences among samples from healthy and diseased plants were tested using Kruskal–Wallis tests and visualized by the “ggplot2” package in R. For the beta diversity, Principal Coordinates Analysis (PCoA) was performed based on the Bray–Curtis distances using the “vegan” package and plotted using the ggplot2 R package. A statistical test of permutational multivariate analysis of variance (PERMANOVA) was carried out using the “vegan” package to see if the microbial community structural composition was consistent across samples, with 999 reciprocals, using the Bray–Curtis distance matrix as input (Guo et al. 2024b). PERMANOVA was also implemented to test the effects of Verticillium wilt disease in a single interval. Random forest analysis was used to quantify the importance of the microorganisms at various taxonomic levels in different groupings (R package “randomForest” (Svetnik et al. 2003)). Differential abundance analysis between the different groups of microbiomes was calculated using EdgeR’s generalized linear model (GLM) approach in “edgeR” R package (Robinson et al. 2010).

## Supplementary Information

Supplementary Material 1.

## Data Availability

The raw sequencing data have been deposited in the Genome Sequence Archive database in National Genomics Data Center, China National Center for Bioinformation, Chinese Academy of Sciences (GSA: CRA034910) that are publicly accessible at https://ngdc.cncb.ac.cn/gsa.

## Abbreviations

HH: Healthy tree branch
DH: Diseased tree-healthy branch
DD: Diseased tree-diseased branch
Hepi: Healthy tree branch epidermis
DHepi: Diseased tree-healthy branch epidermis
DDepi: Diseased tree-diseased branch epidermis
Hxy: Healthy tree branch xylem
DHxy: Diseased tree-healthy branch xylem
DDxy: Diseased tree-diseased branch xylem
ROS: Reactive Oxygen Species
DAB: 3,3’-Diaminobenzidine
CAT: Catalase
POD: Peroxidase
REC: Relative conductivity
MDA: Malondialdehyde
