# Supplementary material for: The bark latent fungus Botryosphaeria dothidea exacerbates branch dieback following the infection with Verticillium dahliae
Source: Stress Biol. 2026 Feb 10;6(1):13. doi: 10.1007/s44154-026-00288-3 (PMC12886702; doi:10.1007/s44154-026-00288-3)
Supplement: Supplementary file 1 — Supplementary Material 1. [file 44154_2026_288_MOESM1_ESM.docx]

- **Supplementary Tables**

**Table S1** *p* values of PERMANOVA tests of bacterial communities

| **group** | **Variation (R^2^)** | **Pr (>F)** |
| --- | --- | --- |
| DDepi/DHepi | 0.175 | **0.001** |
| DDepi/Hepi | 0.142 | **0.003** |
| DHepi/Hepi | 0.109 | **0.011** |
| DDxy/Hxy | 0.075 | 0.084 |
| DDxy/DHxy | 0.085 | **0.008** |
| DHxy/Hxy | 0.072 | 0.179 |

**Table S2** *p* values of PERMANOVA tests of fungal communities

| **group** | **Variation (R^2^)** | **Pr (>F)** |
| --- | --- | --- |
| DDepi/DHepi | 0.510 | **0.001** |
| DDepi/Hepi | 0.438 | **0.001** |
| DHepi/Hepi | 0.204 | **0.002** |
| DDxy/Hxy | 0.554 | **0.001** |
| DDxy/DHxy | 0.591 | **0.001** |
| DHxy/Hxy | 0.101 | 0.145 |

**Table S3** *p* values of PERMANOVA tests of fungal communities excluding *V. dahliae*

| **group** | **Variation (R^2^)** | **Pr (>F)** |
| --- | --- | --- |
| DDepi/DHepi | 0.38628889 | **0.001** |
| DDepi/Hepi | 0.370517165 | **0.001** |
| DHepi/Hepi | 0.203995533 | **0.005** |
| DDxy/DHxy | 0.443223747 | **0.001** |
| DDxy/Hxy | 0.435559651 | **0.001** |
| DHxy/Hxy | 0.101842421 | 0.147 |

**Table S4** Functional annotation of genes related to plant defense-related genes in Cluster 5

| **Gene number** | **Gene ID** | **Gene description** | |
| --- | --- | --- | --- |
| 78 | evm.TU.Chr01.236_evm.TU.Chr01.237, evm.TU.Chr01.2625, evm.TU.Chr01.3236, evm.TU.Chr02.2186, evm.TU.Chr02.2260, evm.TU.Chr02.2327, evm.TU.Chr02.3266, evm.TU.Chr02.3817, evm.TU.Chr02.3819, evm.TU.Chr02.3820, evm.TU.Chr02.3828, evm.TU.Chr02.3846, evm.TU.Chr02.3847, evm.TU.Chr02.3886, evm.TU.Chr02.3922, evm.TU.Chr03.2781, evm.TU.Chr03.2849, evm.TU.Chr03.2872, evm.TU.Chr03.435, evm.TU.Chr04.53, evm.TU.Chr06.2074, evm.TU.Chr06.2525, evm.TU.Chr06.321, evm.TU.Chr06.386, evm.TU.Chr06.395, evm.TU.Chr06.398, evm.TU.Chr06.411, evm.TU.Chr06.413, evm.TU.Chr06.62, evm.TU.Chr06.74, evm.TU.Chr07.126, evm.TU.Chr08.87, evm.TU.Chr09.2334, evm.TU.Chr09.3032, evm.TU.Chr12.28, evm.TU.Chr13.227, evm.TU.Chr13.232, evm.TU.Chr14.1008, evm.TU.Chr14.332, evm.TU.Chr14.333, evm.TU.Chr14.375, evm.TU.Chr14.418, evm.TU.Chr14.586, evm.TU.Chr14.589, evm.TU.Chr14.605, evm.TU.Chr14.835, evm.TU.Chr14.836, evm.TU.Chr14.851, evm.TU.Chr15.373 | NB-ARC, TIR, LRR_8, PAH | |
| 168 | evm.TU.Chr02.202, evm.TU.Chr02.204, evm.TU.Chr02.232, evm.TU.Chr02.240, evm.TU.Chr02.2983, evm.TU.Chr02.2984, evm.TU.Chr02.2985, evm.TU.Chr02.3739, evm.TU.Chr02.3767, evm.TU.Chr03.2850, evm.TU.Chr03.2861, evm.TU.Chr03.3117, evm.TU.Chr03.3118, evm.TU.Chr03.3140, evm.TU.Chr03.3141, evm.TU.Chr03.3143, evm.TU.Chr03.3649, evm.TU.Chr03.529, evm.TU.Chr03.530, evm.TU.Chr03.532, evm.TU.Chr03.536, evm.TU.Chr03.539, evm.TU.Chr03.776, evm.TU.Chr04.1349, evm.TU.Chr04.2802, evm.TU.Chr04.3593, evm.TU.Chr07.1349, evm.TU.Chr08.2785, evm.TU.Chr09.2802, evm.TU.Chr09.2883, evm.TU.Chr10.124, evm.TU.Chr10.2618, evm.TU.Chr11.156, evm.TU.Chr11.1695, evm.TU.Chr14.1729, evm.TU.Chr02.3349, evm.TU.Chr04.1874, evm.TU.Chr04.3393, evm.TU.Chr05.838, evm.TU.Chr13.480, evm.TU.Chr13.656, evm.TU.Chr14.714, evm.TU.Chr14.723, evm.TU.Chr15.2527, evm.TU.Chr10.2311, evm.TU.Chr07.1858, evm.TU.Chr07.1859, evm.TU.Chr07.462, evm.TU.Chr12.1936, evm.TU.Chr13.2715, evm.TU.Chr15.2222 | | LRR_1, LRR_8, B_lectin, LysM, Gal_Lectin, Lectin_legB |
| 86 | evm.TU.Chr02.76, evm.TU.Chr04.1608, evm.TU.Chr04.3375, evm.TU.Chr06.3095, evm.TU.Chr07.1861, evm.TU.Chr07.462, evm.TU.Chr08.261, evm.TU.Chr08.2785, evm.TU.Chr09.2802, evm.TU.Chr10.124, evm.TU.Chr10.2311, evm.TU.Chr10.2649, evm.TU.Chr11.156, evm.TU.Chr11.1695, evm.TU.Chr11.2823, evm.TU.Chr12.1936, evm.TU.Chr13.2715, evm.TU.Chr14.509, evm.TU.Chr14.512, evm.TU.Chr14.696, evm.TU.Chr15.2018, evm.TU.Chr15.2020, evm.TU.Chr15.2022, evm.TU.Chr15.2222, evm.TU.Chr08.3136, evm.TU.Chr03.2260, evm.TU.Chr13.2511, evm.TU.Chr04.2948, evm.TU.Chr08.2771 | | Pkinase, Pkinase_Tyr, Jas, BAG, Response_reg, Hpt |
| 20 | evm.TU.Chr02.2926, evm.TU.Chr02.3432, evm.TU.Chr04.209, evm.TU.Chr04.2118, evm.TU.Chr04.3757, evm.TU.Chr06.103, evm.TU.Chr09.2766, evm.TU.Chr14.136, evm.TU.Chr02.3399, evm.TU.Chr05.52, evm.TU.Chr01.3483, evm.TU.Chr02.2914, evm.TU.Chr03.330, evm.TU.Chr09.179, evm.TU.Chr09.222, evm.TU.Chr05.702, evm.TU.Chr06.2330, evm.TU.Chr07.2917, evm.TU.Chr13.591 | | p450, Terpene_synth, peroxidase, BBE, Dirigent |
| 15 | evm.TU.Chr07.506, evm.TU.Chr12.2690, evm.TU.Chr13.713, evm.TU.Chr01.3328, evm.TU.Chr12.763, evm.TU.Chr02.3699, evm.TU.Chr04.88, evm.TU.Chr05.2460, evm.TU.Chr07.1204, evm.TU.Chr11.2015, evm.TU.Chr12.815, evm.TU.Chr14.640, evm.TU.Chr03.3024, evm.TU.Chr07.543, evm.TU.Chr15.81 | | Chitin_bind_1, Gamma-thionin, Neprosin, Thaumatin, LTP_2, Lung_7-TM_R, PAE |
| 8 | evm.TU.Chr02.3834, evm.TU.Chr02.3839, evm.TU.Chr02.2910, evm.TU.Chr09.2343, evm.TU.Chr04.3379, evm.TU.Chr10.2678, evm.TU.Chr07.2819, evm.TU.Chr07.2821 | | Cellulose_synt, Glucan_synthase, Pectate_lyase_3, Pectinesterase |
| 4 | evm.TU.Chr01.4184, evm.TU.Chr09.6, evm.TU.Chr09.718, evm.TU.Chr01.4184 | | Inhibitor_I29, Inhibitor_I9, Peptidase_C1 |
| 3 | evm.TU.Chr10.243, evm.TU.Chr10.243, evm.TU.Chr10.243 | | Dicer_dimer, Ribonuclease_3, DEAD |
| 12 | evm.TU.Chr03.2260, evm.TU.Chr13.2511, evm.TU.Chr12.44, evm.TU.Chr04.2341, evm.TU.Chr05.2943, evm.TU.Chr13.2184, evm.TU.Chr04.2423, evm.TU.Chr03.209, evm.TU.Chr07.3020, evm.TU.Chr07.67, evm.TU.Chr02.57, evm.TU.Chr06.448 | | BAG, Mlo, Glutaredoxin, Thioredoxin, PUB, AvrRpt-cleavage, MATH, bZIP_1, WRKY |

- **Supplementary Figures**


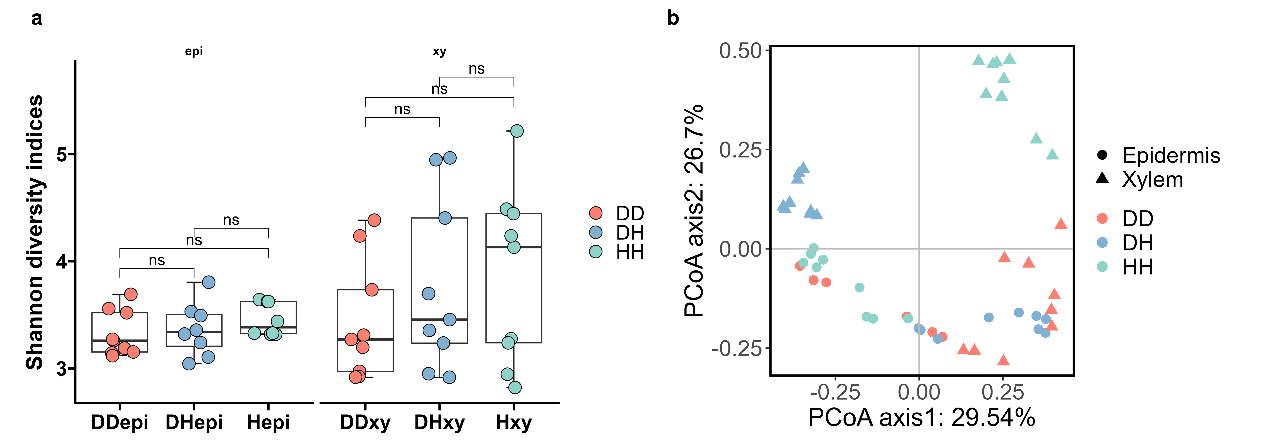


**Fig. S1** Diversity and structure of fungal communities excluding *V. dahliae* in branches and tissue samples from smoke tree (*Cotinus coggygria*). a. Shannon diversity indices of fungi of HH (green), DH (purple) and DD (pink) plants. b. Bray-Curtis distance Principal Coordinate Analysis (PCoA) of fungi.


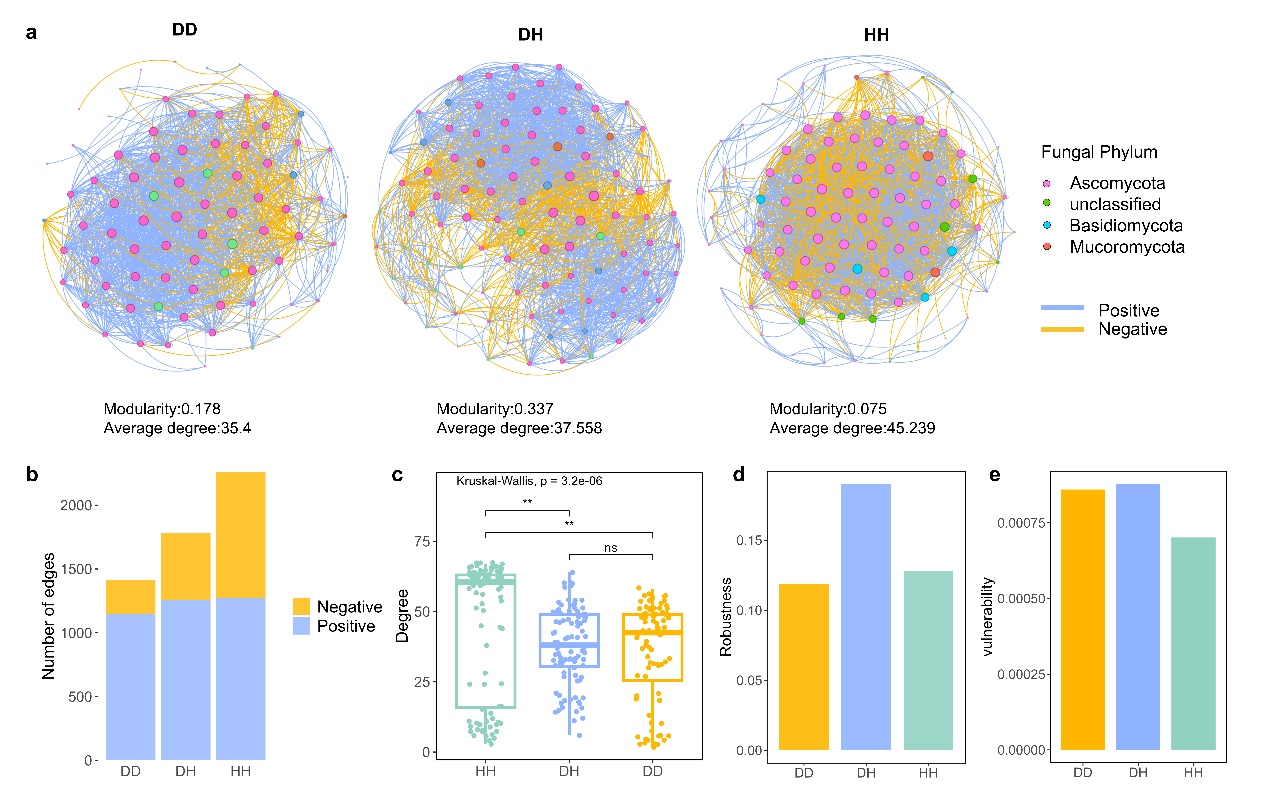


**Fig. S2** Fungal co-occurrence networks of healthy and diseased smoke trees (After *V. dahliae* Removal) **a**. fungal intra-kingdom co-occurrence networks. The nodes are colored according to phylum. Node size indicates the degree of connection. Edge color represents positive (blue) and negative (yellow) correlations. **b**. Edges and of fungal networks. **c**. Degree of fungal networks. **d.** Robustness measured as the proportion of taxa remained with 50% of the taxa randomly removed from each of the fungal networks. **e**. fungal networks vulnerability. Network vulnerability was measured by maximum node vulnerability in each network.


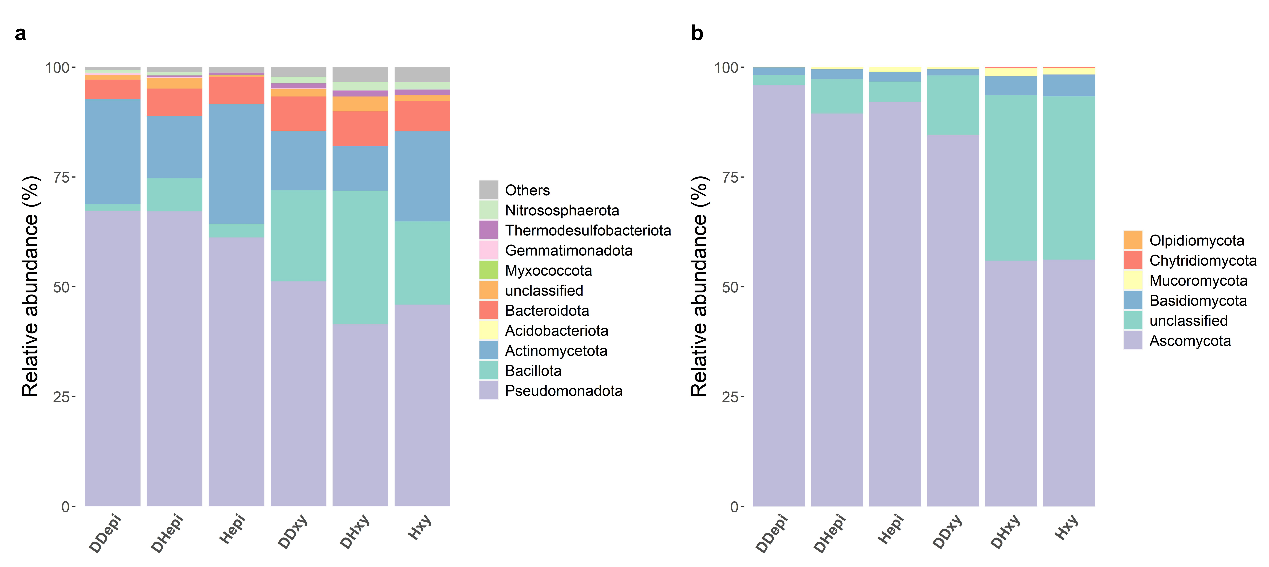


**Fig. S3** Distribution and abundance of major **(a)** bacterial and **(b)** fungal phyla in different compartments of healthy and diseased trees.

**
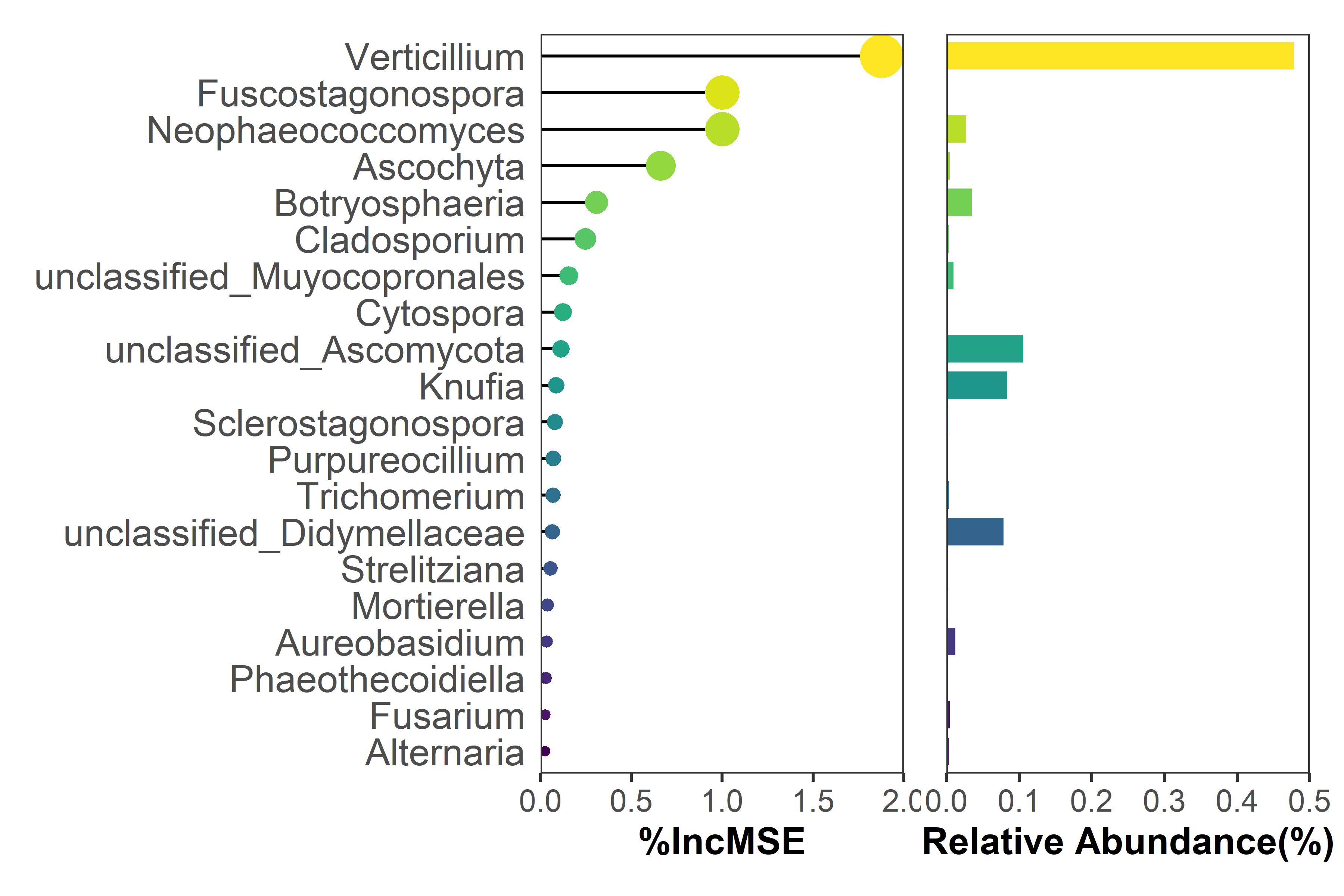
**

**Fig. S4** The top 20 most important fungi biomarkers identified by random-forest classification in the DD and the HH group, with the biomarker taxa ranking in descending order of importance in terms of model accuracy. Mean decrease in Gini was used to evaluate the importance level of fungal classes affected by Verticillium wilt. The bar plot showed the relative abundance of the genus in diseased tree-diseased branches. HH: healthy tree branch; DD: diseased tree-diseased branch.


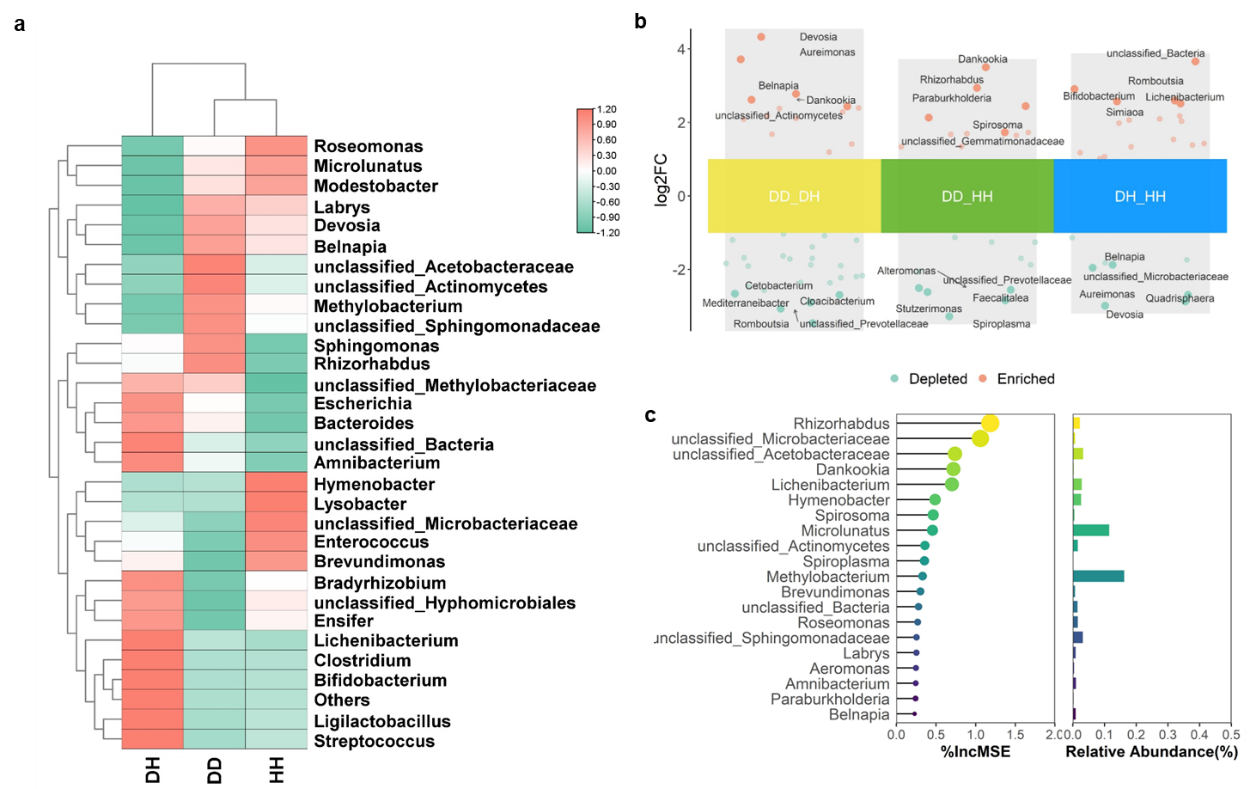


**Fig. S5** Assembly of bacterial communities obtained from smoke tree (*Cotinus coggygria*). **a.** Heat map of the bacterial community composition with cluster analysis. Similar samples were clustered horizontally, and vertical patterns illustrate the phylogenetic relationships among the top 30 genera across samples. **b**. Analysis of the effect of Verticillium wilt on bacterial genus abundance based on volcano plot. Symbols correspond to enrich (red) and deficient (green) genus (P<0.05). **c**. The top 20 most important biomarkers identified by random-forest classification in the DD and the DH group, with the biomarker taxa ranking in descending order of importance in terms of model accuracy. Mean decrease in Gini was used to evaluate the importance level of fungal classes affected by Verticillium wilt. The bar plot showed the relative abundance of the genus in diseased tree-diseased branches. HH: healthy tree branch; DH: diseased tree-healthy branch; DD: diseased tree-diseased branch.


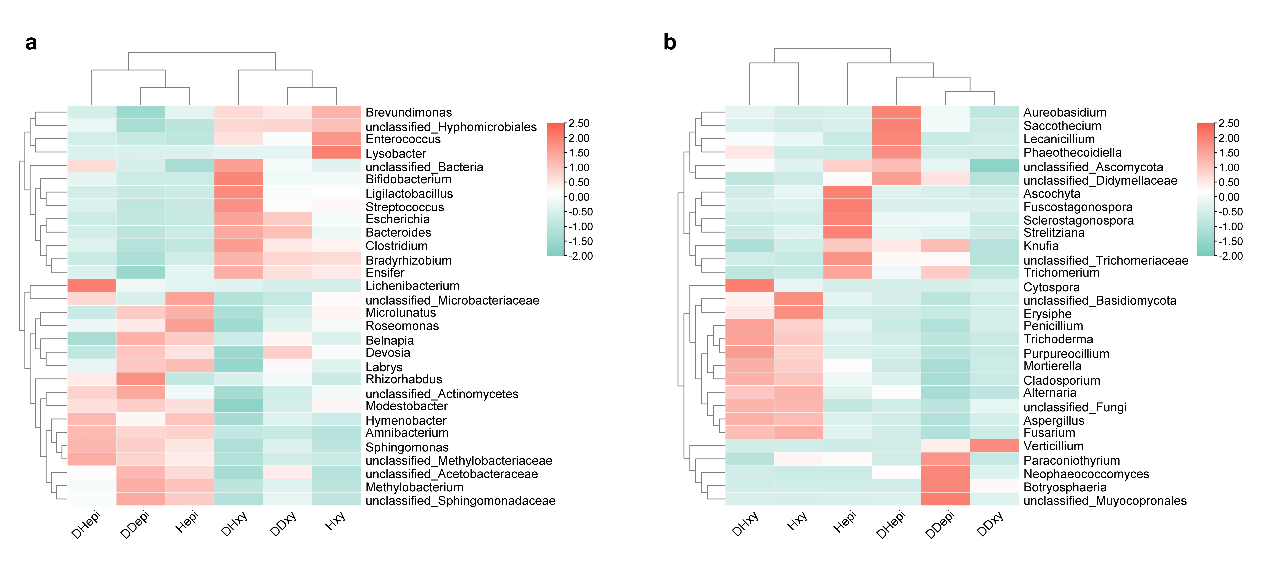


**Fig. S6** Heat map of the bacterial (**a**) and Fungal (**b**)community composition with cluster analysis. Similar samples were clustered horizontally, and vertical patterns illustrate the phylogenetic relationships among the top 30 genera across samples.


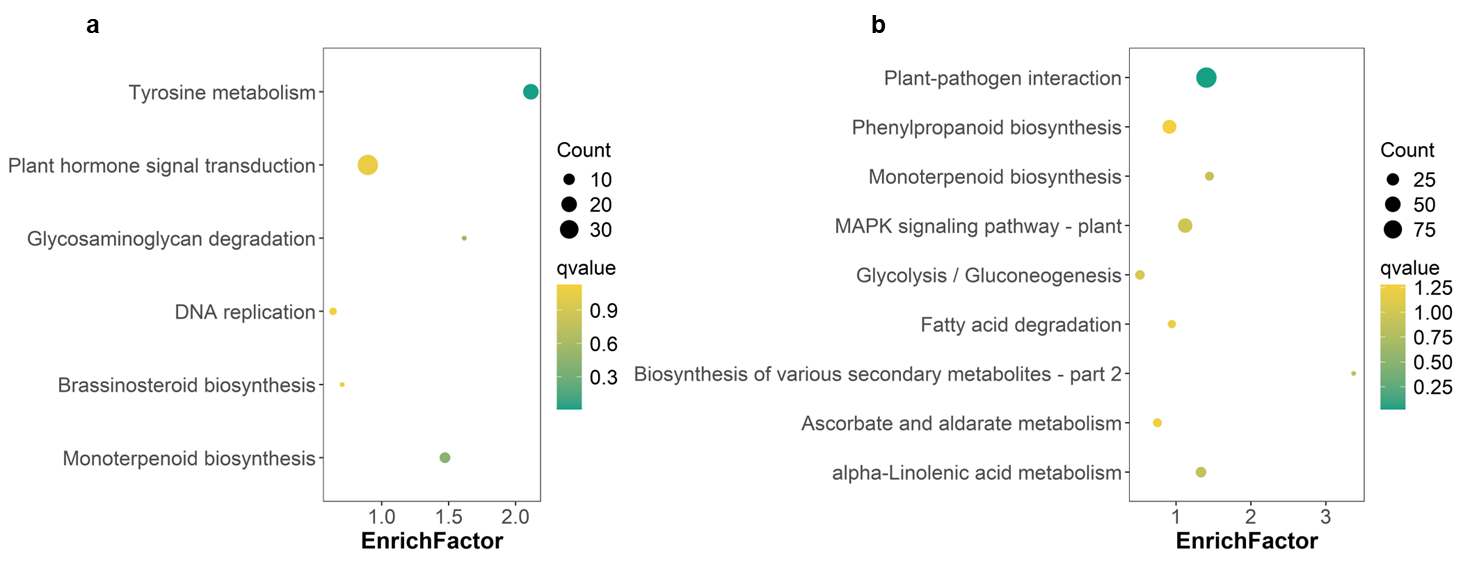


**Fig. S7 Transcriptome profiles of smoke tree branches infected with *Botryosphaeria dothidea.*** KEGG enrichment analysis of downregulated (**a**) and upregulated (**b**) differentially expressed genes. In the graph, the horizontal axis represents the level of significance of pathway enrichment, and the vertical axis represents the KEGG pathways. The size of the dots indicates the number of genes annotated to each KEGG pathway.
